# Photocatalytic degradation of polyethylene films using green-synthesized ZnO and Fe_3_O_4_ nanoparticles from *Acacia nilotica*

**DOI:** 10.1038/s41598-026-43013-w

**Published:** 2026-03-19

**Authors:** Aminu Shaibu, Jimoh Oladejo Tijani, Ambali Saka Abdulkareem, Sherif Ishola Mustapha, Saheed Mustapha, Isaac Alhamdu Baba

**Affiliations:** 1 Department of Molecular Biology and Bioinformatics, Africa Centre of Excellence for Mycotoxin and Food Safety, Federal University of Technology, PMB 65, Gidan Kwano Campus, Minna, Niger State Nigeria; 2 Department of Chemistry, Federal University of Technology, PMB 65, Bosso Campus, Minna, Nigeria; 3 Department of Chemical Engineering, Federal University of Technology, PMB 65, Gidan Kwano Campus, Minna, Niger State Nigeria; 4 Nanotechnology Research Group, Center for Genetic Engineering and Biotechnology, Federal University of Technology, PMB 65, Minna, Niger State Nigeria; 5 Department of Chemical Engineering, University of Ilorin, PMB 1515, Ilorin, Kwara State Nigeria; 6 School of Chemical and Metallurgical Engineering, University of the Witwatersrand, Johannesburg, 2050 South Africa

**Keywords:** Green synthesis, Zinc oxide nanoparticles, Iron oxide nanoparticles, Acacia nilotica, Polyethylene degradation, Photocatalysis, Chemistry, Environmental sciences, Materials science, Nanoscience and technology

## Abstract

This study reports the green synthesis of zinc oxide (ZnO) and iron oxide (Fe_3_O_4_) nanoparticles using *Acacia nilotica* (L.) leaf extract and their application in the photocatalytic degradation of low-density polyethylene (LDPE) and high-density polyethylene (HDPE) films in aqueous medium. Phytochemical screening revealed that *A. nilotica* leaves contained high levels of phenolics (298.21 mg/100 g) and tannins (53.33 mg/100 g), providing strong reducing and stabilizing agents for nanoparticle formation. The synthesized ZnO and Fe_3_O_4_ nanoparticles exhibited UV–Vis absorption peaks at 385.09 and 467.92 nm with corresponding band gaps of 3.22 and 2.65 eV, confirming their semiconducting properties. Dynamic light scattering showed mean particle sizes of 13.64 nm (PDI 0.172) for ZnO and 19.21 nm (PDI 0.309) for Fe_3_O_4_, indicating uniform dispersion and good colloidal stability. XRD and HRSEM analyses confirmed their crystalline, well-defined morphologies, while EDX verified elemental purity. BET surface areas of 216.81 m²/g for ZnO and 199.41 m²/g for Fe_3_O_4_ suggested mesoporosity suitable for catalytic applications. Photocatalytic degradation studies revealed substantial polyethylene breakdown after 30 days under sunlight, with LDPE weight reduced to 73.07% (ZnO) and 74.90% (Fe_3_O_4_), and HDPE reduced to 91.20% and 91.76%, respectively. FTIR spectra of treated films showed the formation of carbonyl, hydroxyl, and C–O functional groups, while SEM images displayed surface cracks and cavities indicative of polymer chain scission. EDX spectra further confirmed Zn and Fe incorporation with intensified oxygen peaks, evidencing oxidative degradation. These findings demonstrate that *A. nilotica*-mediated ZnO and Fe_3_O_4_ nanoparticles are effective and sustainable photocatalysts for polyethylene degradation, offering a promising route toward eco-friendly plastic waste management.

**Supplementary Information:**

The online version contains supplementary material available at 10.1038/s41598-026-43013-w.

## Introduction

The exponential rise in global plastic production has precipitated a major environmental challenge, as conventional polymers such as low-density polyethylene (LDPE) and high-density polyethylene (HDPE) exhibit exceptional resistance to degradation. Their extensive use in packaging, agriculture, and consumer goods has resulted in persistent accumulation in terrestrial and aquatic ecosystems, posing significant ecological and human health risks. Polyethylene, in particular, can persist in the environment for centuries due to its hydrophobic nature, high molecular weight, and limited microbial susceptibility^1^. With plastic consumption projected to continue increasing, the search for sustainable strategies to accelerate polyethylene degradation has become an urgent global priority. Among the emerging solutions, nanotechnology-based photocatalysis has shown considerable promise in breaking down these recalcitrant polymers into simpler and less harmful intermediates^2^.

Conventional nanoparticle synthesis methods, whether chemical or physical, often involve hazardous solvents, elevated temperatures, and toxic by-products, restricting their environmental applicability^3^. Green synthesis has therefore gained prominence as a safer, low-cost, and sustainable alternative. In this approach, plant extracts rich in phytochemicals such as flavonoids, phenolics, tannins, terpenoids, and alkaloids serve as natural reducing and stabilizing agents during nanoparticle formation^4^. This biosynthetic route not only minimizes chemical waste but also produces nanoparticles with enhanced stability and functional activity. Medicinal plants, in particular, have attracted interest due to their diverse bioactive compounds capable of influencing nanoparticle morphology, size, and surface chemistry^5^.

Green synthesis methodologies have recently come to the fore in nanoparticle production as a means of minimizing environmental impact. Conventional chemical and physical synthesis methods often involve the use of hazardous chemicals and/or high energy input and generate toxic by-products that create barriers to sustainability^6,7^. In contrast, plant-mediated green synthesis utilizes bioactive phytochemicals as natural reducing and capping agents that minimize the generation of toxic waste and often result in nanomaterials with improved functional properties. Medicinal and ethnobotanically important plants rich in phenolics, flavonoids, tannins, and other diverse secondary metabolites are of particular value for the synthesis of stable and active nanoparticles^8,9^.

Zinc oxide (ZnO) and iron oxide (Fe_2_O_3_ or Fe_3_O_4_) nanoparticles are among the most extensively studied nanomaterials due to their photocatalytic, antibacterial, and adsorptive properties. ZnO, classified as generally recognized as safe (GRAS), is widely employed in biomedical, agricultural, and environmental applications^10,11^. Under ultraviolet (UV) irradiation, ZnO generates reactive oxygen species (ROS) such as hydroxyl radicals and superoxide ions that effectively degrade persistent organic pollutants, including plastics and dyes^12,13^. Likewise, Fe_3_O_4_ nanoparticles are valued for their magnetic properties, low toxicity, and ability to catalyze Fenton-like reactions that facilitate pollutant oxidation^14^. Combining ZnO and Fe oxide nanoparticles can offer synergistic photocatalytic effects, ZnO primarily driving ROS generation while Fe_3_O_4_ enhances charge separation and electron transfer, thereby improving degradation efficiency in aqueous environments.

The plant species used for nanoparticle synthesis strongly influences the physicochemical characteristics of the resulting materials. *Acacia nilotica* (L.), a medicinal plant abundant in tropical and subtropical regions, is rich in tannins, phenolics, saponins, and flavonoids^15^. These compounds provide both reducing and capping functionalities, improving nanoparticle stability and activity. Although *A. nilotica* extracts have demonstrated antimicrobial, antioxidant, and anti-inflammatory properties, their potential in nanoparticle-mediated plastic degradation remains largely unexplored^16^. Employing *A. nilotica* as a biogenic precursor for ZnO and Fe_3_O_4_ nanoparticle synthesis could therefore provide a sustainable route for producing efficient photocatalysts with minimal environmental footprint.

Photocatalytic degradation of plastics proceeds through light-induced excitation of electrons from the valence to the conduction band of semiconductor nanoparticles, generating electron–hole pairs. These charge carriers interact with water and oxygen to form hydroxyl radicals and superoxide ions, which cleave polymer chains into smaller molecules such as alcohols, aldehydes, and carboxylic acids^17^. The degradation susceptibility of LDPE and HDPE differs due to their structural properties. LDPE’s higher branching and lower crystallinity render it more vulnerable to oxidative attack, whereas HDPE’s denser structure offers greater resistance. Understanding how both polymers respond to nanoparticle-induced photocatalysis is essential for designing efficient remediation systems.

In particular, the classical techniques for dealing with pollutants, which encompass various forms of pharmaceutical treatments or biological treatments, present difficulties for dealing with recalcitrant organic pollutants, particularly hydrophobic polymers, micro-pollutants, and recalcitrant compounds^18,19^. In this regard, advanced oxidation processes (AOPs), which include techniques like photocatalysis, Fenton reactions, electro-Fenton, and ozonation, among others, generate highly potent oxidation species, such as hydroxyl radicals, which have great prospects for dealing with recalcitrant compounds.

Though photocatalytic degradation of organic pollutants, like dyes, phenolic compounds, and pharmaceuticals, has been widely investigated, a low number of attempts were made for polymeric plastics, including green routes for ZnO, Fe₃O₄ nanoparticles, and comparing their activity towards the degradation of LDPE, HDPE^20,21^.

Previous studies have demonstrated the successful plant-mediated synthesis of ZnO and Fe_3_O_4_ nanoparticles for applications in dye removal and antimicrobial treatment^22–25^. However, few investigations have explored their combined use for polyethylene degradation, particularly when synthesized from *A. nilotica* extracts. Bridging this gap is timely, as it integrates green chemistry and environmental nanotechnology to address one of the most persistent forms of plastic pollution.

Therefore, the objectives of the present study are: to successfully green synthesize ZnO and Fe_3_O_4_ nanoparticles using the leaf extract of A. nilotica; to characterize the synthesized nanoparticles for their structural, chemical, and physical properties; and to assess the photocatalytic efficiency of the synthesized nanoparticles for degrading LDPE and HDPE films in aqueous sunlight. This approach to green nanocatalyst formation addresses theoretical, technical, and practical shortcomings in green nanocatalysts to mitigate the menace of plastic pollution afflicting our environment.

## Materials and methods

### Material

Zinc acetate dihydrate (Zn(CH₃COO)₂·2 H₂O, ≥ 99% purity, SigmaAldrich), ferric chloride hexahydrate (FeCl₃·6 H₂O, ≥ 98% purity, SigmaAldrich), ferrous sulfate heptahydrate (FeSO₄·7 H₂O, ≥ 99% purity, SigmaAldrich), sodium hydroxide pellets (NaOH, ≥ 98% purity, Merck), and ethanol (99.5% purity, Merck) were used for the synthesis of nanoparticles. Deionized water was used throughout in all the experiments. All chemicals used were of analytical grade and used as received without further purification.

### Study area

This study was conducted at the Federal University of Technology, Minna (FUTMinna) (supplementary material Fig. S1), located at the Gidan Kwano campus in Minna, Niger State, Nigeria. The site lies between latitudes 9°31′15″N and 9°32′30″N and longitudes 6°26′15″E and 6°28′00″E, approximately 17 km from Minna, the state capital. Minna has an estimated population of 513,000 (Macrotrends, 2024) and is characterized by distinct wet and dry seasons. The region receives annual rainfall ranging from 1,100 to 1,600 mm, with the rainy season spanning from April to October and peaking between June and September. Mean monthly temperatures range from 25.1 °C in August to 30.5 °C in March. These climatic conditions support the growth of diverse tropical plant species used in this study.

### Source, identification, and preparation of plant materials

Fresh and mature leaves of *Calotropis gigantea* (milkweed), *Azadirachta indica* (neem), *Mangifera indica* (mango), and *Acacia nilotica* (L.) (acacia) were collected from the FUTMinna Gidan Kwano campus, Niger State, Nigeria. Plant identification and authentication were performed by experts in the Department of Plant Biology, Federal University of Technology, Minna. Before collection, leaves were carefully inspected to ensure they were healthy and disease-free. The harvested leaves were transported to the ACEMFS Laboratory for further processing. They were thoroughly washed under running tap water to remove dust and impurities, air-dried at room temperature for two weeks, and then ground into a fine powder using an electric blender. The powdered samples were stored in airtight containers at 4 °C until further phytochemical analysis as detailed in the supplementary material (Sect.  2.3 S1.2-2.3 S6).

#### Extraction for phytochemical analysis

Aqueous extraction of the plant materials was performed following the procedure described by Ayyanar et al.^26^ with slight modifications. Briefly, 10 g of the powdered leaf sample of each plant species (*C. gigantea*, *A. indica*, *M. indica*, and *A. nilotica*) was mixed with 100 mL of deionized water (1:10 w/v). The mixtures were stirred intermittently and allowed to stand for 48–72 h at room temperature to facilitate the extraction of phytochemicals. The resulting suspensions were filtered using sterile Whatman No. 1 filter paper to obtain clear aqueous extracts. Each filtrate was stored in a clean, labeled container and refrigerated at 4 °C until further use for phytochemical screening and nanoparticle synthesis.

### Polymer samples and pre-treatment

Commercial low-density polyethylene (LDPE) and high-density polyethylene (HDPE) shopping bags were used as the model polyolefin films. Characterization and experiments were initiated after the polymer films were cut into standardized square shapes measuring 1.5 × 1.5 cm. The average initial mass for the individual films was set at (LDPE: 50 mg, HDPE: 50 mg), and the range for the films was narrow. Film thickness was in the range of 25–35 μm for LDPE and 30–40 μm for HDPE, based at various points for each film type assessed with a digital micrometer. The polymers were employed without treatment; however, no such grade-specific details of the manufacturer were available. It has been acknowledged that there might be traces of general processing additives, such as antioxidants, slip agents, or stabilizers, that can exist in polyethylene films of commercial grade. In order to minimize the effect of surface contaminants, inks, or loosely bound additives with which the samples may be contaminated during handling, a pre-treatment of all the samples before analysis was carried out. All the samples were washed with a mixture of ethanol/water in sequence, followed by drying under ambient conditions, followed by drying at 50 °C to constant weight.

Additives, crystallinity, or film morphology have been identified to affect polyethylene photostability, though for the purposes of the present work, comparative trends in degradation are being studied under controlled conditions identical to those already accepted by established polymeric property structures for photodegradation phenomena.

### Gravimetric analysis and mass-loss determination

The changes in the masses of the polymers were evaluated gravimetrically, with the initial absolute masses (mg) before treatment, as well as the final absolute masses (mg) after treatment, recorded for the LDPE samples, as well as the HDPE samples. The samples were carefully retrieved, rinsed with deionized water, then oven-dried at a controlled 50 °C, where the cycles were undertaken in a repeating drying-weighing process to the point where the samples reached a constant weight, which in this study was taken as the difference where the variation in the masses was below ± 0.2 mg in the last two consecutive cycles, with the calculation for both the absolute amount of weight changed (mg) as well as the percentage amount (%).

Detached polymer fragments present in the aqueous phase were not quantitatively recovered or filtered before weighing. Therefore, the measured mass loss was a result of the combined effects of chemical degradation, physical fragmentation, as well as the loss of loosely bound oxidized products. Similar procedures for rinsing, drying, and weighing were carried out for all the test samples as well as the controls to avoid any selected or systemic bias.

### Green synthesis of ZnO and Fe₃O₄ nanoparticles using *Acacia nilotica* (L.) extract

#### Synthesis of ZnO nanoparticles

**Fig. 1:**
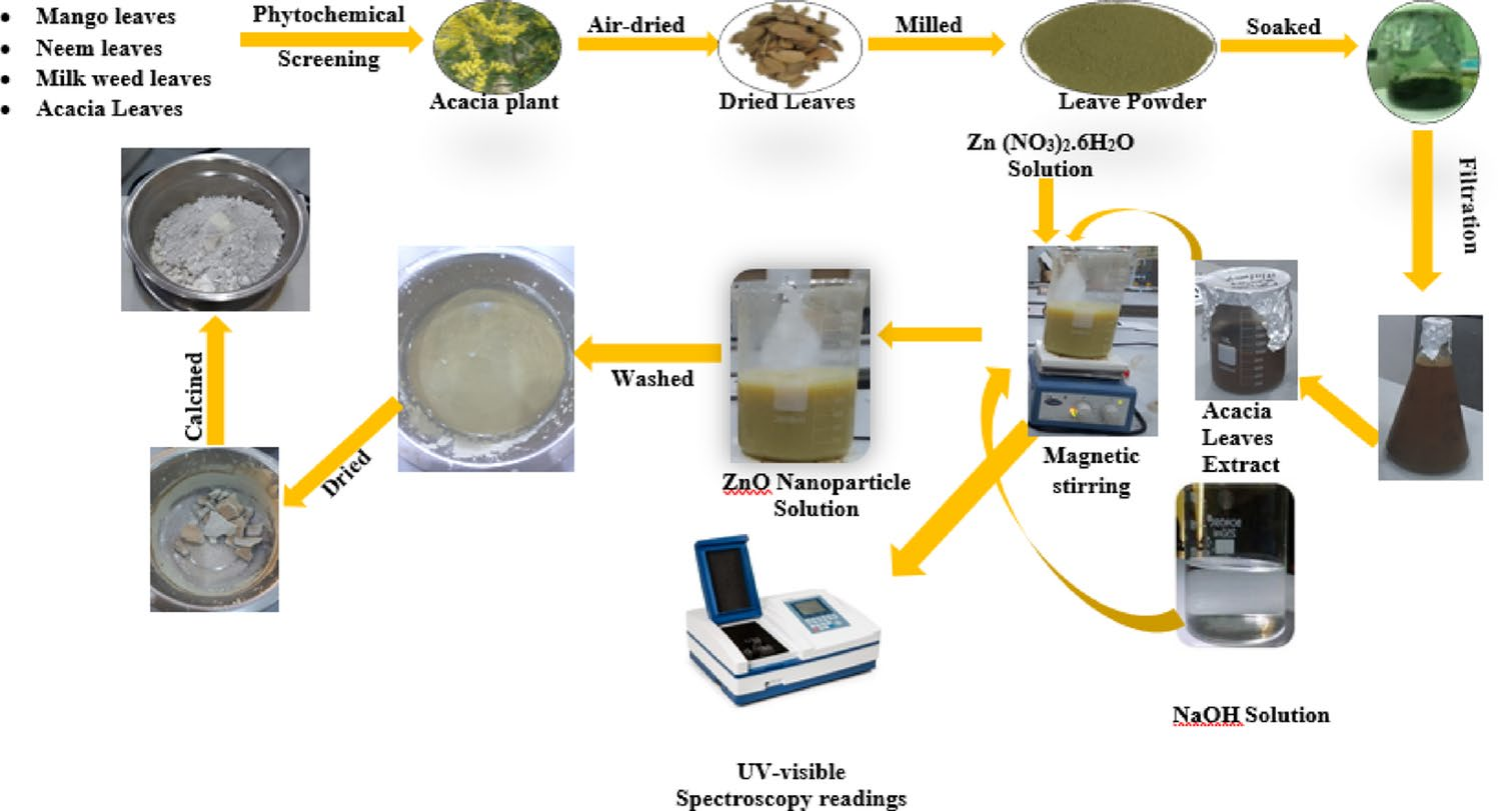
Schematic representation of the synthesis of zinc oxide (ZnO) nanoparticles.

Zinc oxide nanoparticles (ZnO NPs) were synthesized following a modified method described by Abdelbaky et al.^27^. A 14.87 g quantity of zinc nitrate hexahydrate (Zn(NO_3_)_2_·6H_2_O) was dissolved in 50 mL of deionized water under continuous stirring to prepare a 1 M solution. After complete dissolution, 50 mL of *Acacia nilotica* (L.) aqueous leaf extract was added dropwise to the zinc nitrate solution and stirred for 15 min. The pH of the mixture was adjusted to 10 using sodium hydroxide (NaOH) until a cream-colored suspension formed, indicating nanoparticle precipitation. The reaction was maintained under constant stirring for an additional 45 min to ensure complete conversion. The resulting precipitate was centrifuged (Xiangtian Model 800-1, 1790 × g) at 4000 rpm for 10 min and washed several times with deionized water to remove residual impurities. The purified precipitate was dried in an oven at 90 °C overnight and subsequently calcined at 450 °C for 2 h to obtain a fine white ZnO nanoparticle powder. The schematic steps involved in the green synthesis of ZnO nanoparticles are illustrated in Fig. 1.

#### Synthesis of Fe₃O₄ nanoparticles

**Fig. 2:**
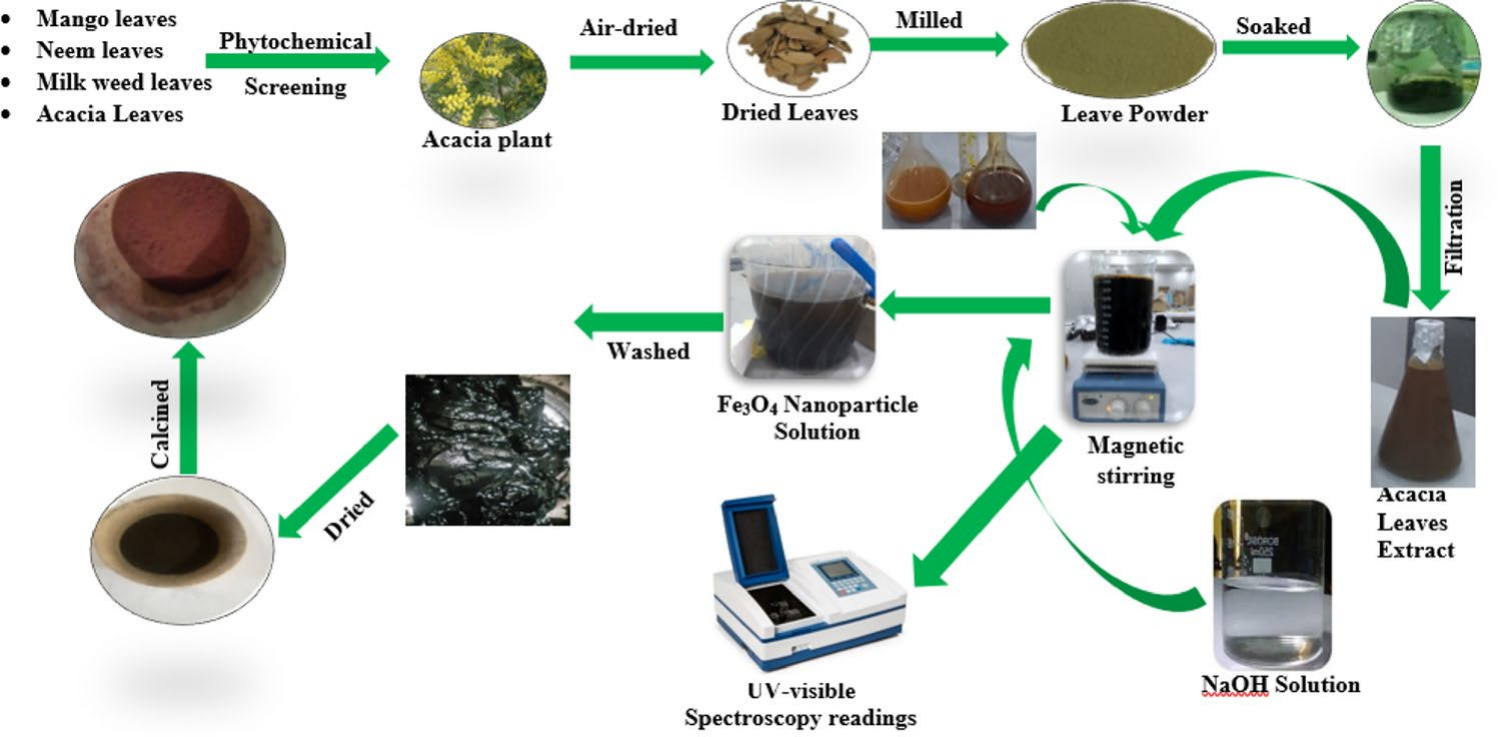
Schematic representation of the synthesis of magnetite (Fe_3_O_4_) nanoparticles.

Iron oxide nanoparticles (Fe_3_O_4_ NPs) were synthesized using a green route adapted from Yusefi et al.^28^. Separate 1 M solutions of iron(II) chloride tetrahydrate (FeCl_2_·4H_2_O) and iron(III) chloride hexahydrate (FeCl_3_·6H_2_O) were prepared by dissolving 4.06 g and 3.17 g of each salt, respectively, in 25 mL of deionized water. The two solutions were then mixed in a 2000 mL beaker under magnetic stirring for 15 min to ensure homogeneity. Following this, 50 mL of *Acacia nilotica* (L.) aqueous leaf extract was added gradually to the mixture under constant stirring. The pH was adjusted to 10 using NaOH, upon which the solution turned black, indicating the formation of Fe_3_O_4_ nanoparticles. Stirring was continued for an additional 45 min to allow for complete reaction and nanoparticle precipitation. The resulting black precipitate was centrifuged, washed repeatedly with deionized water, and dried at 90 °C overnight. The dried material was calcined at 450 °C for 2 h to yield a fine dark-brown Fe_3_O_4_ nanoparticle powder. The schematic steps for Fe_3_O_4_ nanoparticle synthesis are shown in Fig. 2.

### Characterization of zinc oxide (ZnO) and magnetite (Fe_3_O_4_) nanoparticles

The synthesized nanoparticles were characterized to evaluate their structural, morphological, and optical properties. X-ray diffraction (XRD) was used to determine crystallinity, while Fourier-transform infrared spectroscopy (FTIR) identified functional groups involved in nanoparticle formation. Morphological and elemental analyses were carried out using high-resolution scanning electron microscopy (HRSEM) coupled with energy-dispersive X-ray spectroscopy (EDS). Particle size, distribution, and surface charge were analyzed through dynamic light scattering (DLS), and surface area and porosity were determined using the Brunauer–Emmett–Teller (BET) technique. Optical properties and band gaps were analyzed using UV–Vis spectroscopy. The detailed methodology is in **Sect.  2.7 S1-2.7 S6** of the supplementary material.

### Photocatalytic degradation of LDPE and HDPE using ZnO and Fe_3_O_4_ nanoparticles

For photocatalytic degradation experiments, low-density polyethylene (LDPE) and high-density polyethylene (HDPE) film specimens were sourced from commercially packaged plastics in the form of small plastic bags, such as the one used by Kumar et al.^29^. The plastic specimens were cut into small films with dimensions 1.5 × 1.5 cm and weighed with high precision before degradation experiments. Each plastic film sample was suspended in a 10-mL suspension medium consisting of deionized water and 1 g/L zinc oxide (ZnO) nanoparticles and iron oxide (Fe₃O₄) nanoparticles or deionized water in a separated set of experiments under similar conditions but without nanoparticles, and in completely dark conditions with total shading from light.

A photocatalyst shining under sunlight was implemented during a 30-day interval in September 2024 in Minna, Nigeria (9.6° N, 6.6° E). The photostability was conducted outside under unobstructed open sky during the sun-rising interval from 09:00 to 18:00 h under average sunlight exposure of 9 h.day⁻¹. However, direct measurements of sun irradiance, such as UV irradiance and total irradiance in W · m⁻², were not available to determine the sun exposure on a surface area. Moreover, the available meteorological data for Minna during the month of September suggests that solar intensity is always high since daily UV index values range from “moderate” to “very high” (5 to 10), and the atmospheric conditions are mostly “clear” to “partly cloudy.” All the reactors stood at the same height and orientation.

The biological experiments were conducted using sealed borosilicate glassware, while gentle constant agitation of the suspensions at around 100 rpm using an orbital shaker, as well as exposure during daylight h, prevented nanoparticle settlement while allowing for homogenous interaction of the plastic film, photocatalytic nanoparticles, and water medium^30–32^. The solution temperature was not controlled but followed ambient conditions, keeping it between 27 and 35 °C, which is an average temperature during the course of the daytime, as all samples were exposed simultaneously.

The pH of each sample of suspension was measured at 5-day intervals in order for chemical changes in the degradation process. Both test and control films were collected at each sampling interval, gently washed in deionized water, and finally oven-dried for several 3–4-hour sessions under 50 °C conditions until constant weight was achieved. The percentage weight loss of the films was used as an indicator of degradation efficiency and calculated using Eq. (1):

1 $$\mathrm{Weight\:loss\:(\%)}=\frac{{W}_{i}-{W}_{f}}{{W}_{i}}\times100$$

where *Wi* and *W*_*f*_ represent the initial and final film weights, respectively.

In addition to weight loss analysis, structural and chemical transformations were evaluated. Fourier-transform infrared spectroscopy (FTIR) was employed to identify the formation of new oxygenated functional groups (e.g., carbonyl, hydroxyl, and C–O), indicative of oxidative chain scission. Scanning electron microscopy (SEM) was used to examine surface morphological alterations, including cracks, voids, and roughness, enabling comparison between nanoparticle-treated and control films^33^.

### Mineralization assessment via CO₂ evolution

In determining whether photocatalytic-induced oxidative degradation of polyethylene proceeded to mineralization or was confined to surface oxidation, CO₂ production was taken into account, which stands out as one of the more prominent indicators of mineralization. CO₂ production has been taken as the ultimate fate of carbon present in a polymer, rendered into carbon dioxide, generally viewed as definitive of degradation rather than surface processes^30,34^.

#### Experimental design for monitoring CO₂ evolution

Photocatalytic mineralization experiments were conceptually set up in a closed reactor system having a sealed borosilicate glass chamber (250 mL), equipped with a gas-tight septum. The LDPE and HDPE foils (1.5 × 1.5 cm) were placed in aqueous mixtures containing ZnO or Fe₃O₄ nanoparticles (density: 1 g L⁻¹) under identical sunlight exposure as discussed in Sect.  2.9. Control experiments were also carried out: (i) absence of nanoparticles and (ii) in darkness.

The headspace of the reactor was sampled periodically via a gas-tight syringe, and the concentration of CO₂ was analyzed either by:

- Alkali trapping followed by titrimetric analysis, where evolved CO₂ was absorbed in a standardized NaOH solution and quantified by back-titration with HCl.

#### Quantification of mineralization degree

To estimate the amount of carbon dioxide (CO₂) produced in the photocatalytic treatment of LDPE and HDPE films, an alkali-trapping technique and a back titration technique were used. In this technique, the gas samples drawn intermittently through a closed system reaction vessel headspace were passed into a certain volume of standardized NaOH solution, in accordance with Eq. (2):

2 $${CO}_{2}+2NaOH\to{Na}_{2}{CO}_{3}+{H}_{2}O$$

The remaining NaOH after absorption was determined by back titration against standardized HCl using phenolphthalein as the indicator. The quantity of NaOH consumed by CO₂ was therefore the difference between the initial NaOH moles and the remaining NaOH moles that were obtained from titration. Considering that two moles of NaOH react stoichiometrically with one mole of CO₂, the number of moles of CO₂ (${n}_{C{O}_{2}}$) evolved is calculated using Eq. (3):

3 $${n}_{C{O}_{2}}=\frac{{C}_{NaOH}{V}_{NaOH}\times{C}_{HCl}{V}_{HCl}}{2}$$

${C}_{NaOH}$ and ${V}_{NaOH}$ are the concentration and volume of NaOH used for CO₂ trapping, and ${C}_{HCl}$ and ${V}_{HCl}$ are the concentration and volume of HCl consumed during back-titration.

The degree of mineralization (M, %) was calculated based on cumulative CO₂ evolution relative to the initial carbon content of the polyethylene sample using Eq. (4):

4 $$\mathrm{M}\left(\boldsymbol{\%}\right)=\frac{{n}_{{CO}_{2}}\times12}{{M}_{c,polymer}}\times100$$

where.

${\boldsymbol{n}}_{{\boldsymbol{C}\boldsymbol{O}}_{2}}$ is the number of moles of CO₂ evolved,

12 is the molar mass of carbon (g mol⁻¹), and.

${M}_{c,polymer}$​ is the initial mass of carbon present in the polyethylene film.

The calculation was based on the assumption of an approximate carbon content of 86% for LDPE and HDPE. The accumulated CO_2_ evolution was calculated by adding up the CO_2_ evolved at various time intervals after subtracting the results of the control experiments performed without nanoparticles and in the dark.

This quantitative approach of CO_2_ allows a distinct differentiation to be made between the surface-level oxidative degradation and the actual mineralization of polyethylene carbon into CO_2_. This will offer a more accurate evaluation of the photocatalysis ability than the weight loss and spectroscopic variation methods.

### Data analysis

All experimental analyses were conducted in triplicate, and results were expressed as mean ± standard deviation. Data were statistically analyzed using the Statistical Package for the Social Sciences (SPSS, Version 23). One-way analysis of variance (ANOVA) was performed to determine significant differences among treatments, and Duncan’s Multiple Range Test (DMRT) was used for post hoc comparison of means at *p* < 0.05. Percentage calculations were performed using Microsoft Excel 2016, and graphical and tabular representations were prepared to illustrate the findings.

## Results and discussion

### Phytochemical screening

Table 1 summarizes the concentrations of key bioactive compounds (phenols, flavonoids, tannins, saponins, and alkaloids) present in the leaves of four plant species: *Acacia nilotica* (L.) (acacia), *Calotropis gigantea* (calotropis), *Mangifera indica* (mango), and *Azadirachta indica* (neem). Among the tested plants, *A. nilotica* exhibited the highest levels of phenolic compounds (298.21 mg/100 g) and tannins (53.33 mg/100 g), along with appreciable quantities of saponins. The elevated phenolic and tannin contents of *A. nilotica* are particularly significant, as these phytochemicals play a crucial role in the bioreduction and stabilization of metal ions during nanoparticle synthesis. Phenolic hydroxyl groups act as electron donors, facilitating the reduction of metal precursors to their corresponding oxides, while tannins enhance nanoparticle capping and prevent agglomeration through complexation and surface binding interactions^35^.

Although *M. indica* also contained relatively high phenolic and saponin levels, its lower flavonoid concentration may have limited its overall reducing capacity. In contrast, *C. gigantea* exhibited comparatively higher alkaloid content, which, while bioactive, contributes less effectively to the rapid reduction of metal ions. These observations are consistent with reports by Barathikannan et al.^4^ and Abbas et al.^5^, who noted that phenol- and tannin-rich plant extracts are superior reducing agents for green nanoparticle synthesis. Therefore, *A. nilotica* was selected as the optimal candidate for synthesizing zinc oxide (ZnO) and iron oxide (Fe_3_O_4_) nanoparticles in this study, owing to its abundance of strong reducing and stabilizing phytochemicals. This choice aligns with the principle that phenol-dominant plant extracts promote the formation of uniformly dispersed stable nanoparticles with enhanced catalytic performance.

**Table 1:** Phytochemical screenings of plant leaf extract.

Plant leaves	Phenols (mg/100 g)	Flavonoids (mg/100 g)	Tannins (mg/100 g)	Saponins (mg/100 g)	Alkaloids(mg/100 g)
*Acacia nilotica (L)* (Acacia)	298.21^a^ ± 0.65	19.94^b^ ± 0.62	53.33^a^ ± 0.32	89.77^b^ ± 0.45	9.92^b^ ± 0.60
*Calotropis gigantea* (Linn.) Aiton F.	173.21^d^ ± 0.70	16.73^c^ ± 0.26	36.83^d^ ± 0.62	65.12^d^ ± 0.73	13.43^a^ ± 0.42
*Mangifera indica* (Mango)	291.94^b^ ± 0.75	14.99^c^ ± 0.65	49.73^b^ ± 0.61	95.53^a^ ± 0.68	8.99^b^ ± 0.67
*Azadirachta indica* (Neem)	196.65^c^ ± 1.12	43.76^a^ ± 0.53	45.88^c^ ± 0.53	74.69^c^ ± 0.81	12.26^a^ ± 0.61

Data are represented in triplicate in the table as Mean ± standard deviation. Values with different superscripts in a column are significantly different (*p* ≤ 0.05).

#### UV–Vis analysis of ZnO and Fe_3_O_4_ nanoparticles

**Fig. 3:**
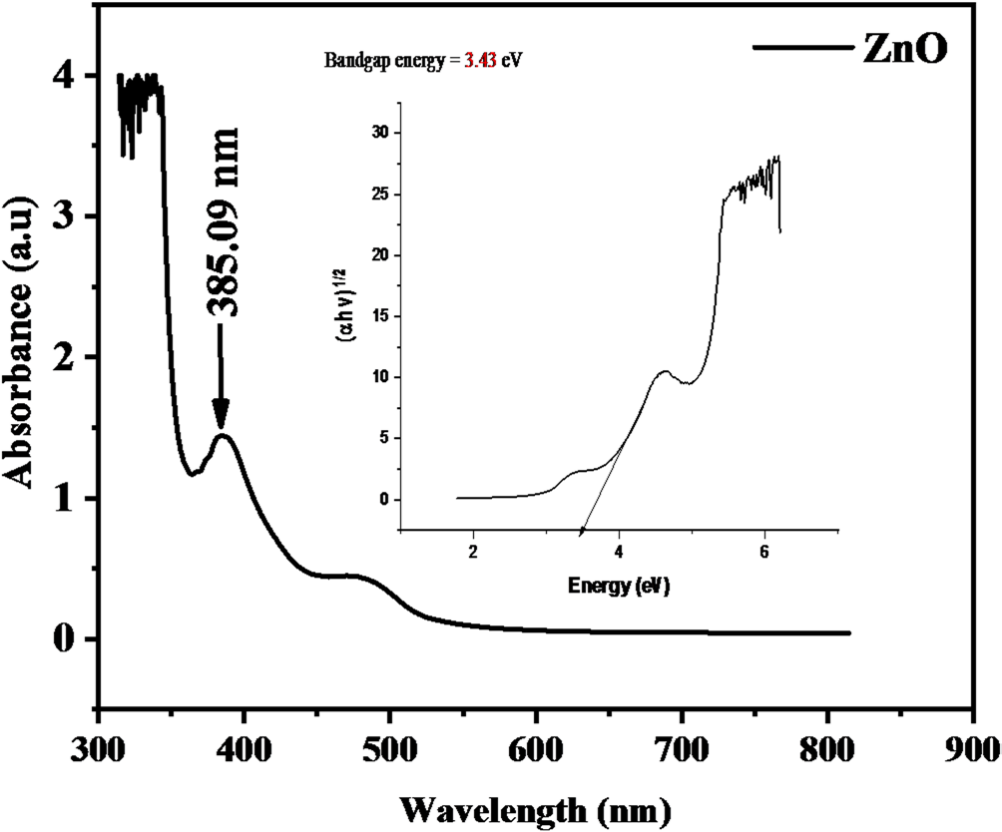
UV–Vis spectrum and corresponding band gap energy of ZnO nanoparticles.

The optical properties of the synthesized ZnO and Fe_3_O_4_ nanoparticles were analyzed using UV–Visible spectroscopy. Figure 3 shows the UV–Vis absorption spectrum of ZnO nanoparticles, which exhibited a distinct and sharp absorption peak at 385.09 nm within the UV region (200–400 nm). The corresponding band gap energy, determined from the Tauc plot, was 3.22 eV, consistent with the typical range of 3.2–3.3 eV reported for ZnO nanoparticles^36,37^. The pronounced peak indicates efficient electronic transitions from the valence band to the conduction band, confirming the semiconducting nature of ZnO. The rapid decline in absorbance beyond 400 nm suggests that ZnO primarily absorbs ultraviolet radiation while transmitting visible light, a property desirable for photocatalytic applications under sunlight. The sharpness of the peak further reflects the uniform particle size and crystallinity of the ZnO nanoparticles, which contribute to enhanced charge separation efficiency and optical stability^38^. The strong UV absorption capability underscores the potential of ZnO nanoparticles for use in photocatalytic degradation of persistent organic pollutants and plastic waste under solar irradiation^39^.

**Fig. 4:**
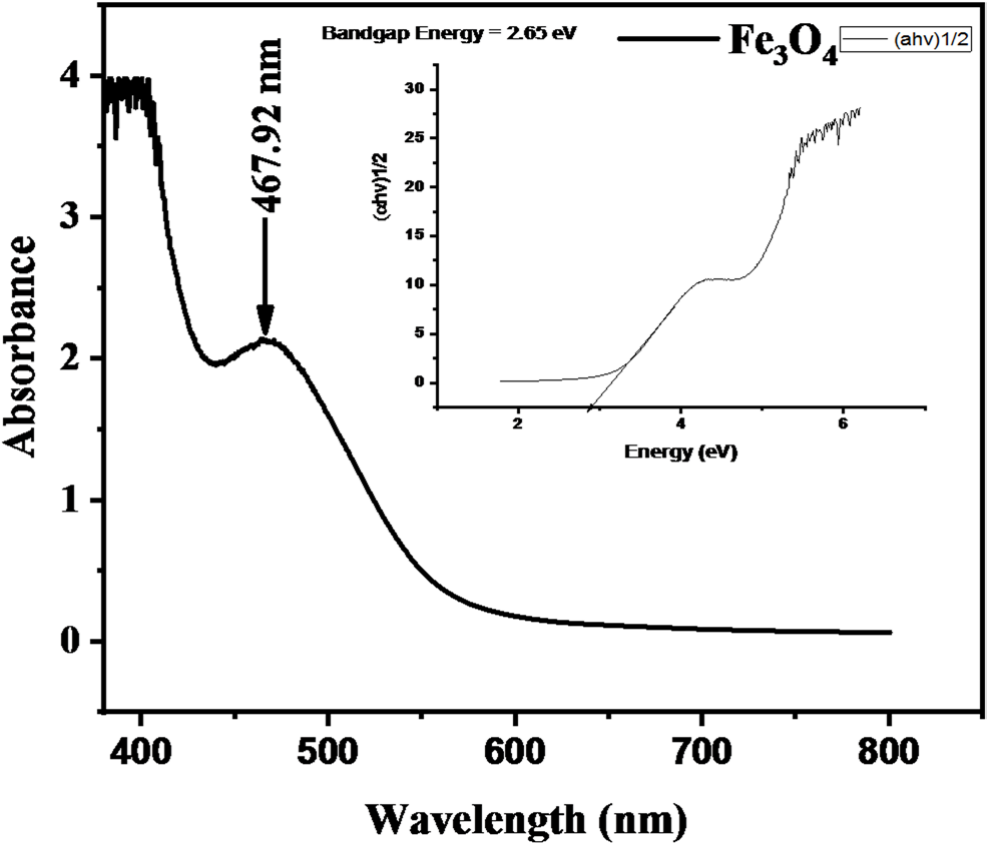
UV–Vis spectrum and corresponding band gap energy of Fe_3_O_4_ nanoparticles.

The UV–Vis absorption spectrum of Fe_3_O_4_ nanoparticles is presented in Fig. 4. A prominent absorption peak was observed at 467.92 nm. The optical band gap, calculated from the Tauc plot, was 2.65 eV, within the reported range of 1.76–2.95 eV for magnetite nanoparticles^40^. This moderate band gap value suggests that the synthesized Fe_3_O_4_ nanoparticles exhibit semiconductor-like behavior, enabling photoactivation under UV and near-visible light. Furthermore, band gap estimates were undertaken cautiously because of differences in the band gap determination of diffuse reflectance vs. absorbance of suspensions of the particles, which involved consideration of the transition that was considered applicable; band gap estimates using the Tauc equation proved to be based on the complex nature of the compound’s valence band structure of Fe_3_O_4_^41^. The steady decrease in absorbance beyond 500 nm indicates limited light interaction in the visible range, implying good optical selectivity. These properties enhance Fe_3_O_4_’s applicability in photocatalytic and bioremediation systems, where partial transparency to visible light allows effective light penetration to the reaction surface^42–44^. The observed spectral stability also indicates strong Fe–O bonding and a well-defined crystal structure, essential for maintaining photocatalytic efficiency during extended sunlight exposure.

The UV–Vis results confirm the successful biosynthesis of optically active ZnO and Fe_3_O_4_ nanoparticles using *Acacia nilotica* extract. Their band gap energies (3.22 eV for ZnO and 2.65 eV for Fe_3_O_4_) indicate favorable semiconductor characteristics suitable for solar-driven photocatalytic degradation of polyethylene films.

#### Dynamic light scattering (DLS) analysis of ZnO and Fe_3_O_4_ nanoparticles

**Fig. 5:**
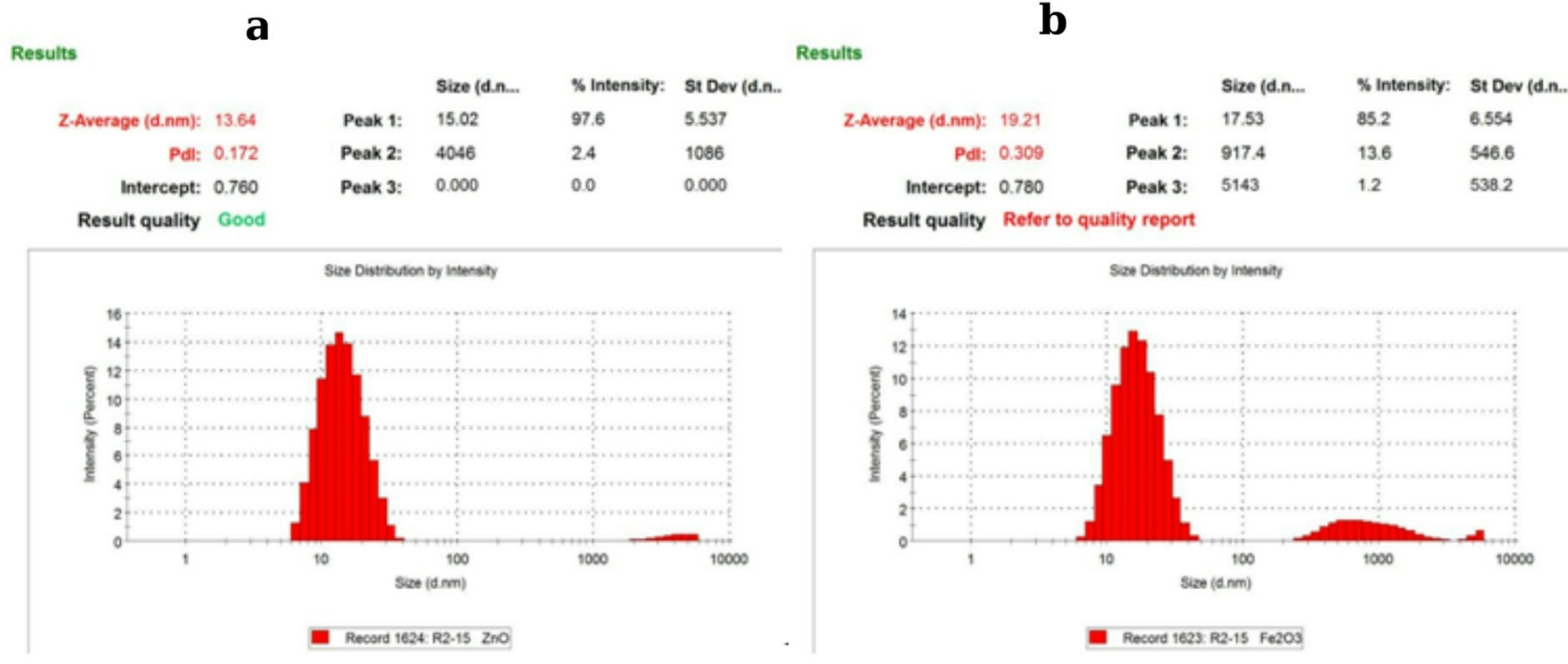
DLS size distribution profiles of (**a**) ZnO and (**b**) Fe_3_O_4_ nanoparticles.

The samples for DLS measurements of the metal oxide nanoparticles were dispersed in aqueous medium at a concentration of 1 mg mL⁻¹, measured at pH 7 with ionic strength in the low mM range (< 10 mM) to balance signal quality and colloidal behavior. The particle size distribution and colloidal stability of the synthesized ZnO and Fe_3_O_4_ nanoparticles were evaluated using dynamic light scattering (DLS), as shown in Fig. 5. For ZnO nanoparticles (Fig. 5a), the particle size ranged from 13.64 to 55.57 nm, with a Z-average diameter of 13.64 nm and a dominant size peak at 11.96 nm, accounting for 99.9% of the total volume. The low polydispersity index (PDI = 0.172) indicates a narrow size distribution and high monodispersity, confirming uniform particle formation. The small particle size and homogeneity suggest that the *Acacia nilotica* extract effectively acted as both a reducing and capping agent, limiting agglomeration and promoting consistent nucleation. These nanoscale dimensions significantly enhance the specific surface area, improving light absorption and photocatalytic efficiency, key parameters for applications in UV absorption, environmental remediation, and biocatalysis^45^. For Fe_3_O_4_ nanoparticles (Fig. 5b), particle sizes ranged from 19.21 to 41.72 nm, with a Z-average diameter of 19.21 nm and a predominant size peak near 17.53 nm. The PDI value of 0.309 reflects a moderately narrow distribution, indicative of a stable colloidal suspension with minimal aggregation. Although slightly higher than that of ZnO, this PDI remains within the acceptable range for magnetite nanoparticles, where mild magnetic interactions can promote limited clustering. The observed particle uniformity confirms that the phytochemicals in *A. nilotica* extract effectively stabilized the Fe_3_O_4_ nanoparticles, preventing extensive aggregation while preserving their nanoscale morphology^45^. The relatively small size and moderate dispersity of Fe_3_O_4_ nanoparticles also make them advantageous for magnetic separation and photocatalytic applications, particularly in environmental systems involving microplastic and polymer degradation. The DLS results validate that *A. nilotica*-mediated synthesis produced well-dispersed ZnO and Fe_3_O_4_ nanoparticles with strong colloidal stability, ideal for subsequent photocatalytic studies.

### X-ray diffraction (XRD) analysis of ZnO and Fe_3_O_4_ nanoparticles

**Fig. 6:**
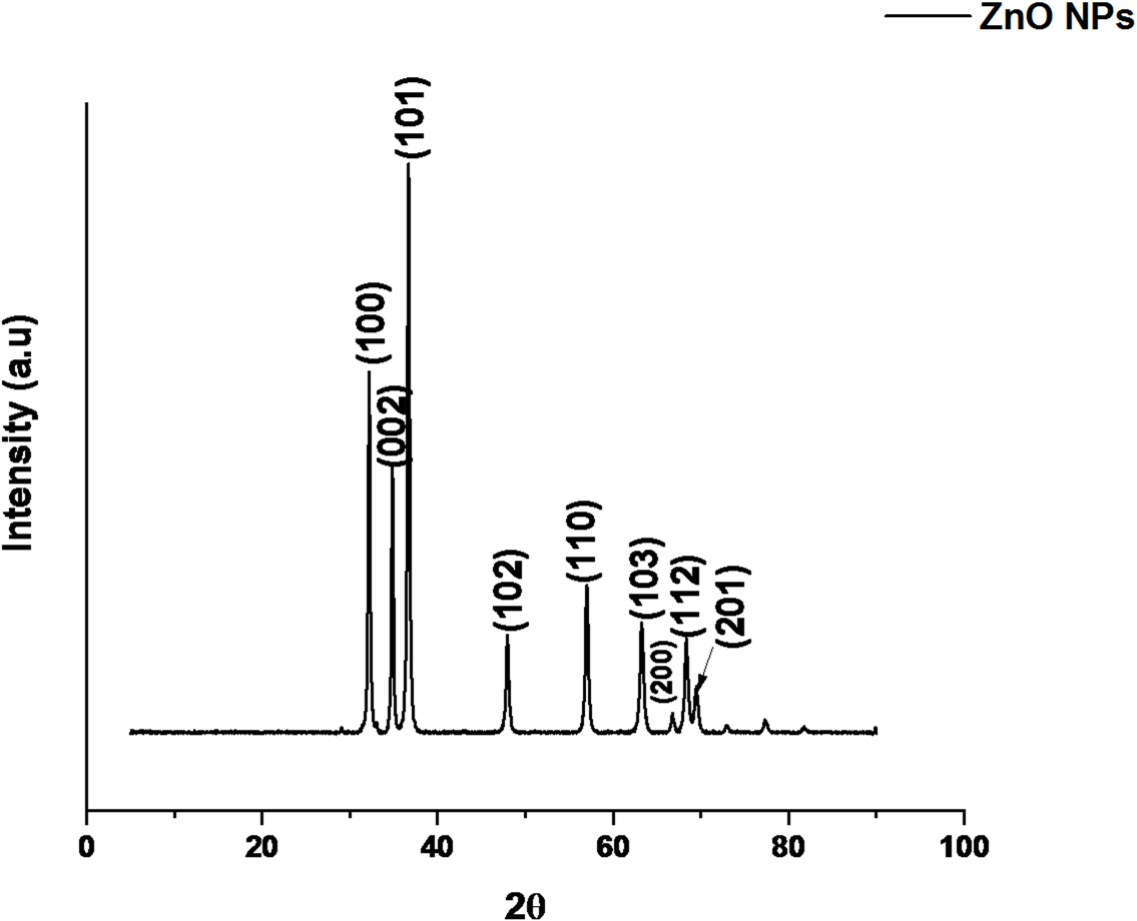
XRD pattern of green-synthesized ZnO nanoparticles.

**Fig. 7:**
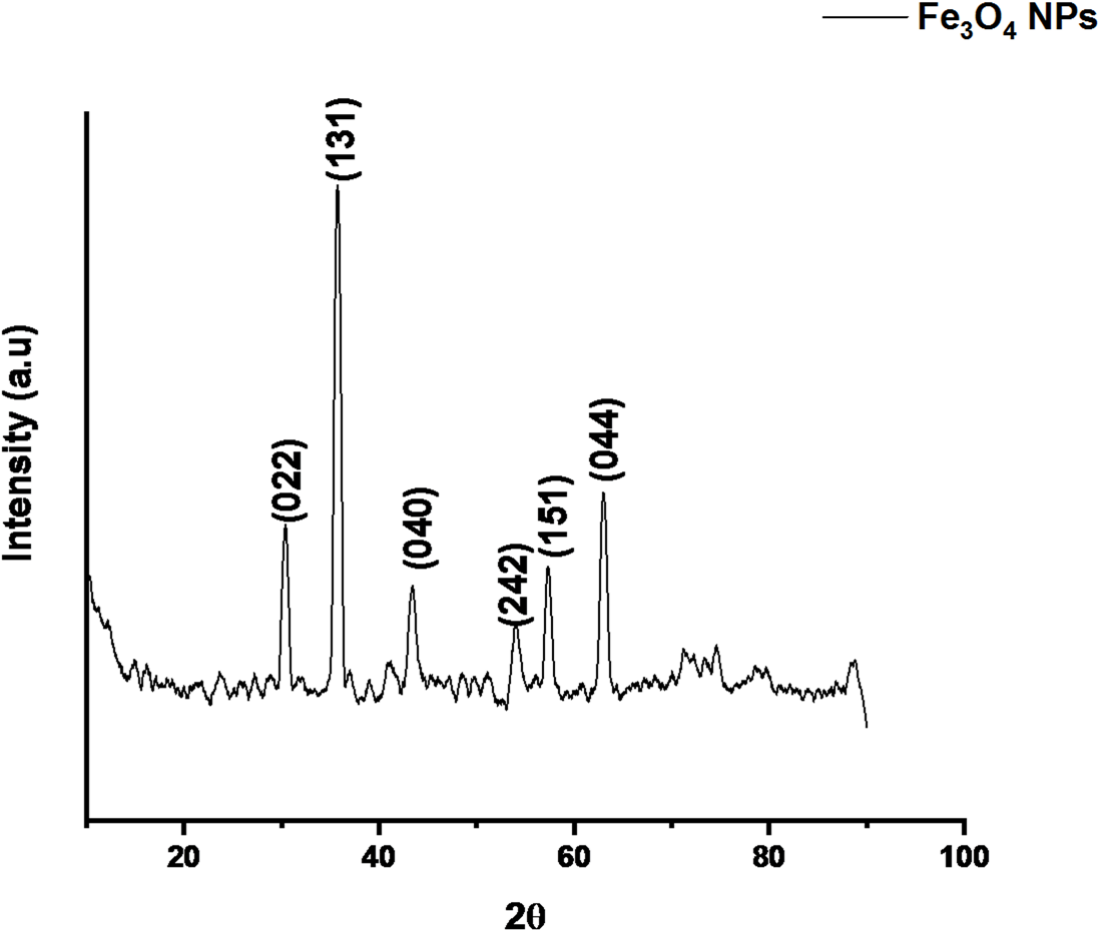
XRD pattern of green-synthesized Fe_3_O_4_ nanoparticles.

The crystalline structures of the synthesized ZnO and Fe_3_O_4_ nanoparticles were characterized using X-ray diffraction (XRD), and the diffraction patterns are shown in Figs. 6 and 7, respectively. For ZnO nanoparticles (Fig. 6), distinct diffraction peaks were observed at 2θ values of 31.90°, 34.68°, 36.80°, 47.79°, 56.63°, 63.81°, 66.67°, 68.28°, and 69.38°, corresponding to the (100), (002), (101), (102), (110), (103), (200), (112), and (201) crystal planes. These reflections match well with the Joint Committee on Powder Diffraction Standards (JCPDS) card no. 00-005-0664, confirming the formation of a hexagonal wurtzite ZnO structure. The sharp and intense peaks indicate a high degree of crystallinity, while the absence of impurity peaks suggests phase purity. The average crystallite size, calculated using the Debye–Scherrer equation, was approximately 17.68 nm, consistent with values reported for plant-extract-mediated ZnO nanoparticles^46–48^. The small crystallite size is attributed to the capping and stabilizing effects of phytochemicals (phenols and flavonoids) in *Acacia nilotica* extract, which effectively regulate particle nucleation and growth. The high crystallinity and nanoscale dimension of ZnO are advantageous for enhanced photocatalytic performance in the degradation of polymeric contaminants.

Similarly, the Fe_3_O_4_ nanoparticles exhibited characteristic diffraction peaks at 2θ values of 30.09°, 35.44°, 43.05°, 53.38°, 56.97°, and 62.53°, corresponding to the (220), (311), (400), (422), (511), and (440) planes of magnetite (Fig. 7). These peaks are well indexed to the cubic spinel structure of Fe_3_O_4_ as per JCPDS card no. 96-900-5842. The calculated average crystallite size was 46.17 nm, which falls within the typical 30–70 nm range reported for green-synthesized Fe_3_O_4_ nanoparticles^49,50^. The broadening of diffraction peaks suggests the presence of minor lattice strain and a narrow crystallite size distribution, commonly observed in biosynthesized magnetic nanoparticles. The absence of secondary or impurity peaks confirms the high phase purity of the product. This structural purity and crystalline uniformity contribute to the material’s magnetic stability and photocatalytic activity, which are critical for applications such as plastic degradation and wastewater treatment^51^.

### HRSEM and EDX analysis of ZnO and Fe_3_O_4_ nanoparticles

#### HRSEM and EDX of ZnO nanoparticles

**Fig. 8:**
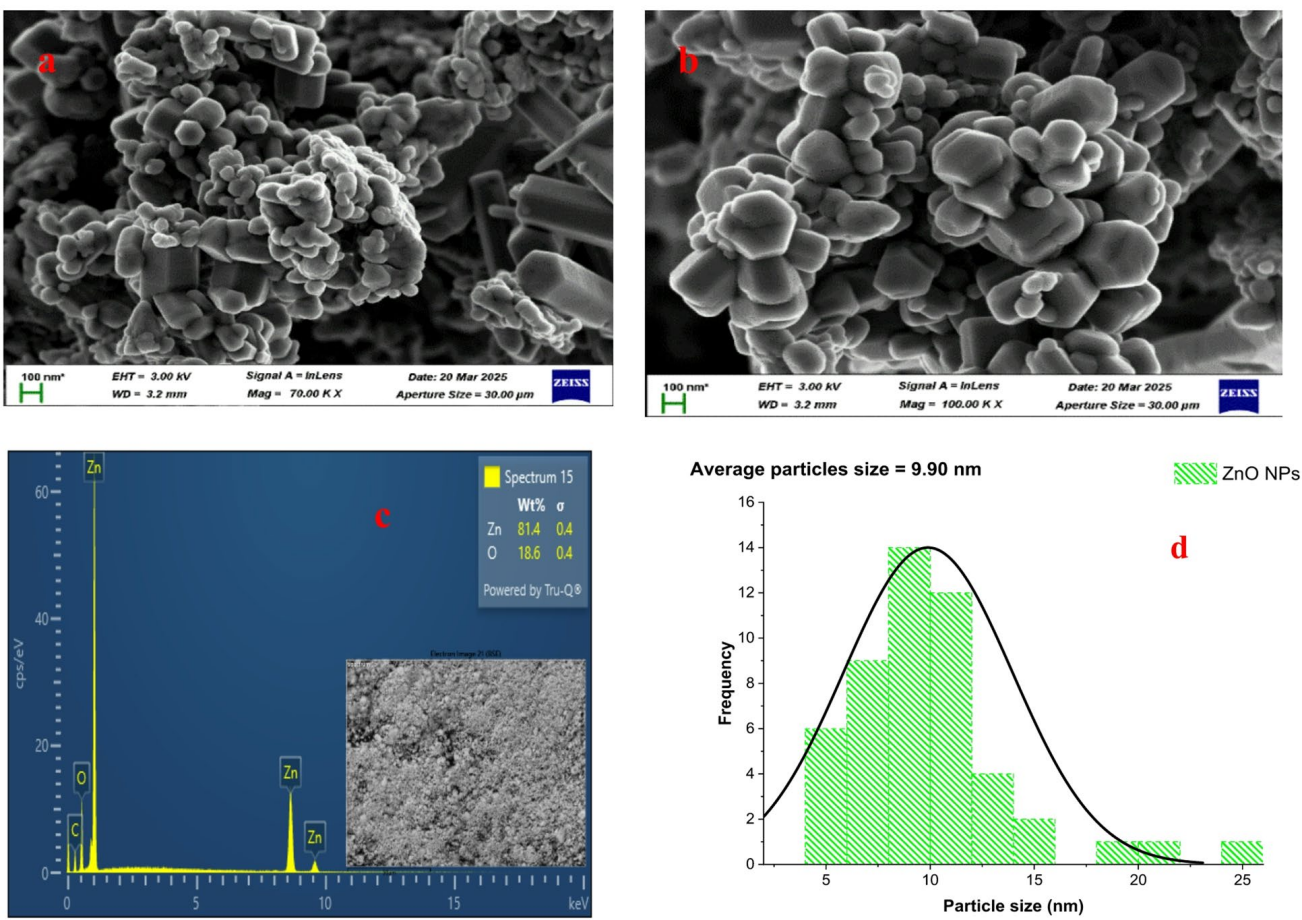
(**a**–**b**) HRSEM micrographs, (**c**) EDX spectrum, and (**d**) particle size distribution of green-synthesized ZnO nanoparticles.

The HRSEM micrographs of the green-synthesized ZnO nanoparticles (Fig. 8a–b) reveal the formation of hexagonal plate-like nanostructures with uniform morphology and minimal agglomeration. The well-defined hexagonal arrangement is typical of wurtzite ZnO crystals, indicating that the synthesis process using *Acacia nilotica* leaf extract successfully yielded highly crystalline nanostructures. The homogeneous distribution and compact clustering of particles suggest strong control over nucleation and growth, attributed to the capping effect of phytochemicals such as phenols and tannins present in the plant extract.

The surface morphology exhibits densely packed nanoplates with sharp edges, offering an extended surface area that enhances photocatalytic reactivity. The average particle size estimated from HRSEM micrographs using ImageJ software was approximately 9.90 nm (Fig. 8d), which falls well within the nanoscale range (0–100 nm). This value is smaller than the crystallite size obtained from XRD analysis (17.68 nm), a difference expected since XRD measures internal crystalline domains while HRSEM assesses the external morphology. Comparable nanostructures have been reported by Ismail et al.^47^ and Khao et al.^48^, confirming the reliability of plant-mediated synthesis for producing high-quality ZnO nanoparticles. The EDX spectrum (Fig. 8c) further confirmed the composition of the nanoparticles, showing only zinc (Zn) and oxygen (O) peaks at approximately 1 keV and 8–9 keV for Zn and around 0.5 keV for O. Quantitative analysis revealed 81.4 wt% Zn and 18.6 wt% O, consistent with the stoichiometric composition of ZnO. The absence of any impurity peaks validates the high purity of the synthesized nanoparticles. Similar purity levels have been reported by Alberti et al.^38^ and Wang et al.^52^, who emphasized that the use of plant extracts in green synthesis prevents contamination and enhances the photocatalytic properties of ZnO for polymer degradation.

#### HRSEM and EDX of Fe_3_O_4_ nanoparticles

**Fig. 9:**
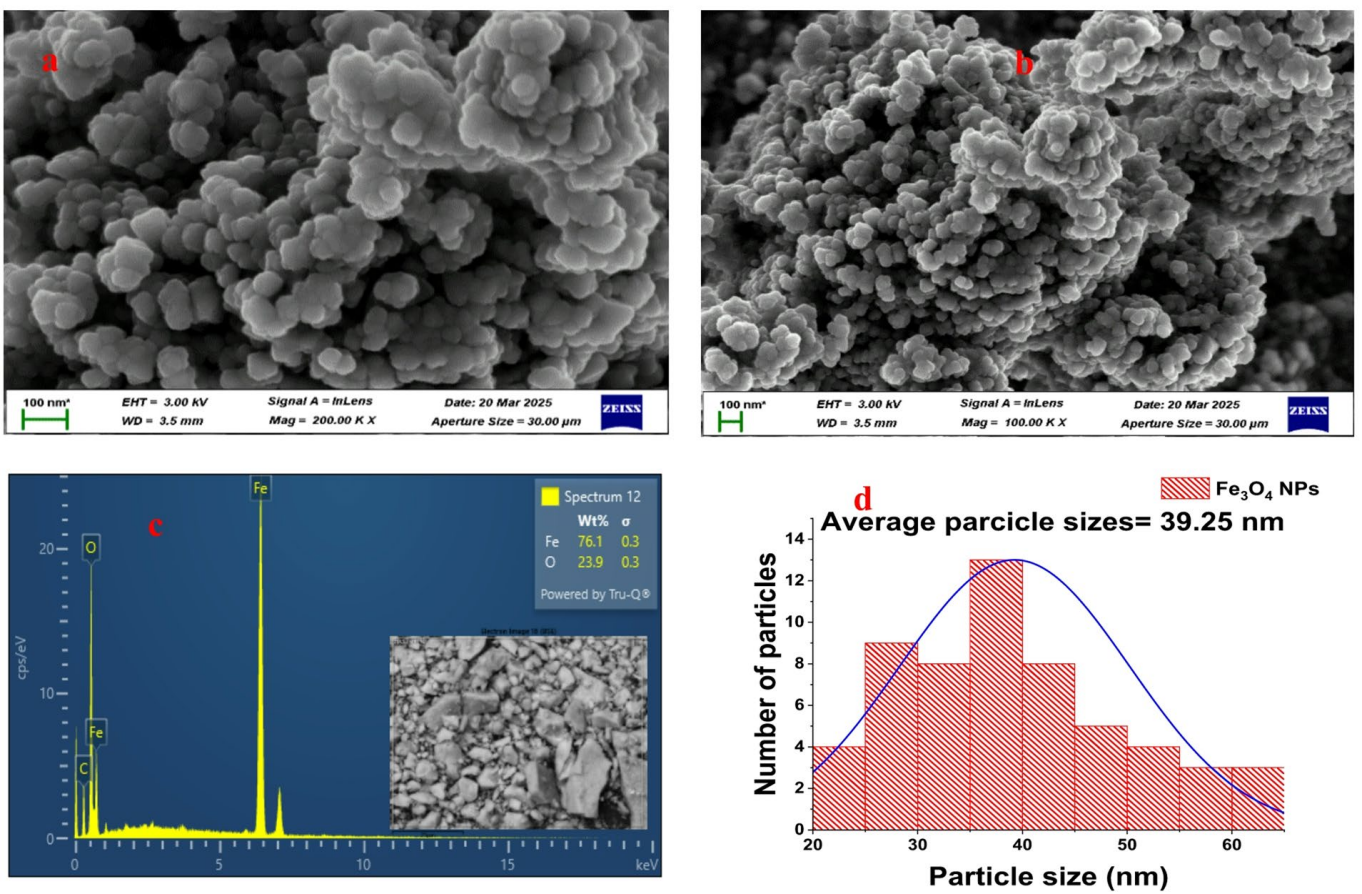
(**a**–**b**) HRSEM micrographs, (**c**) EDX spectrum, and (**d**) particle size distribution of green-synthesized Fe_3_O_4_ nanoparticles.

The HRSEM images of Fe_3_O_4_ nanoparticles (Fig. 9a–b) show irregularly shaped particles with a predominantly cubic morphology, randomly distributed and closely packed with slight agglomeration. This aggregation is typical of magnetic materials such as magnetite, resulting from interparticle magnetic attraction and van der Waals forces. The observed cubic structure aligns with the XRD findings, confirming the formation of highly crystalline Fe_3_O_4_ nanoparticles. The porous surface morphology evident in the images suggests potential adsorption sites capable of trapping polymer fragments during degradation. ImageJ-based analysis (Fig. 9d) revealed that the nanoparticles ranged from 20 to 65 nm, with an average particle size of 39.25 nm. These dimensions are within the reported range for biosynthesized Fe_3_O_4_ nanoparticles^53,54^. The slight variation in size and moderate aggregation can be attributed to the magnetic nature of Fe_3_O_4_ and the influence of synthesis pH, which facilitates the formation of Fe(OH)_2_ intermediates that subsequently convert to Fe_3_O_4_^55^. Despite minor clustering, the nanoparticles retained uniformity and stability, reflecting the stabilizing role of *Acacia nilotica* phytochemicals during synthesis.

The EDX spectrum (Fig. 9c) confirmed the elemental purity of the Fe_3_O_4_ nanoparticles, showing only strong peaks for iron (Fe) and oxygen (O), indicative of Fe–O bond formation characteristic of magnetite. The absence of other elemental impurities highlights the effectiveness of the green synthesis approach in producing chemically pure Fe_3_O_4_ nanoparticles. Similar findings were reported by Lu et al.^56^, who demonstrated that plant-extract-mediated synthesis yields magnetite nanoparticles with high phase purity and enhanced catalytic performance for environmental remediation.

#### XPS of ZnO nanoparticles

**Fig. 10:**
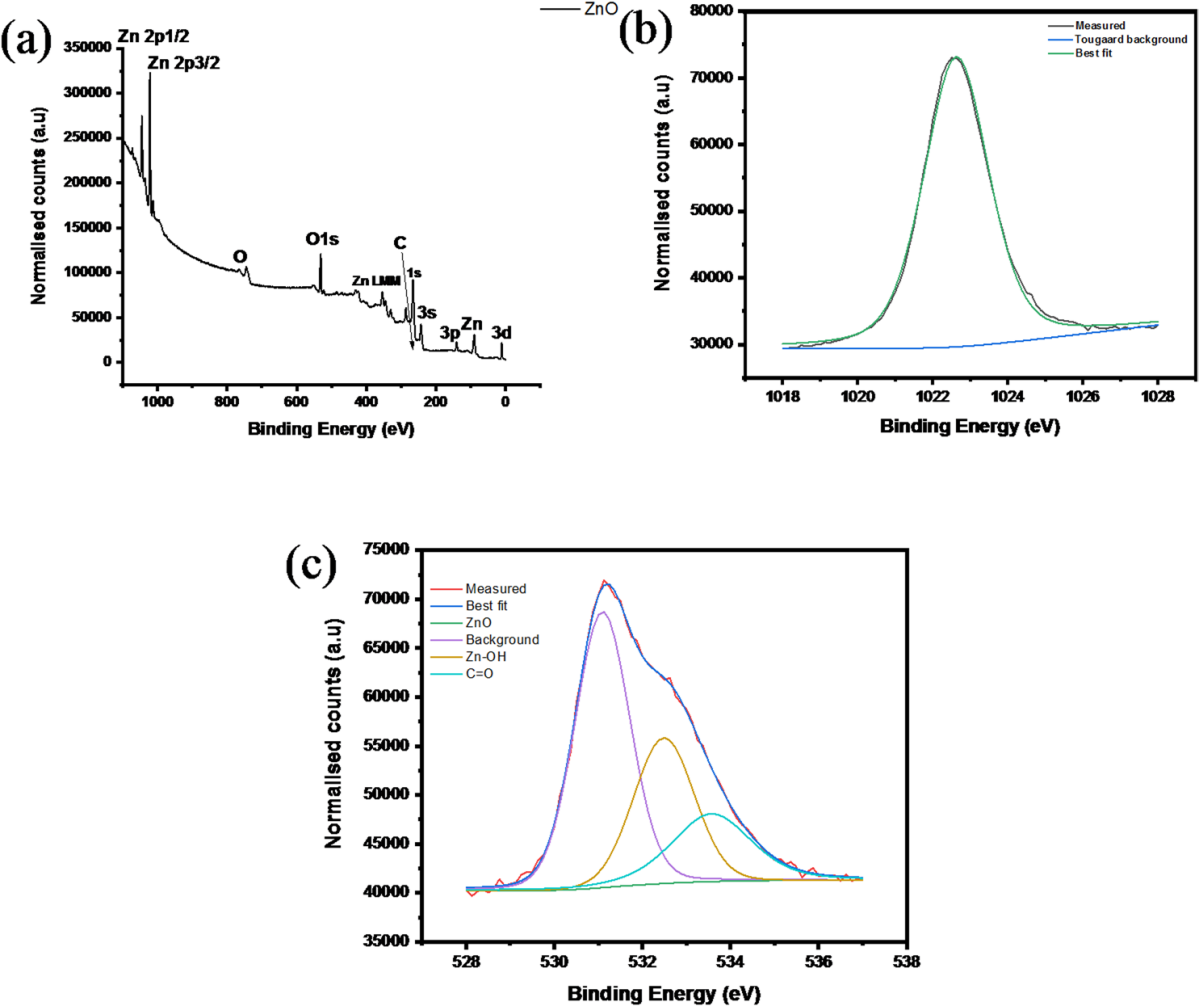
XPS spectra (**a**) Survey of ZnO, (**b**) ZnO, (**c**) Oxygen.

The XPS results offer conclusive proof of the successful green synthesis and surface chemistry of the ZnO nanoparticles. As can be seen from the survey spectrum (Fig. 10(a)), the presence of Zn and O in abundance confirms the formation of ZnO, while the small peak of C 1s is due to adventitious carbon or the presence of trace amounts of plant-derived phytochemicals used as capping agents, which is a common observation in green-synthesized nanomaterials. The high-resolution spectrum of Zn 2p (Fig. 10**(b)**) consists of two distinct and symmetric peaks representing Zn 2p₃/₂ and Zn 2p₁/₂, which appear at binding energies characteristic of Zn²⁺ in ZnO, with a spin-orbit splitting of about 23 eV, thus ruling out the possibility of the presence of metallic Zn or zinc suboxide^34^.

The O 1s peak (Fig. 10**(c)**) can be resolved into several components, with the dominant peak assigned to lattice oxygen (O²⁻) bonded to Zn in the wurtzite ZnO crystal structure, while the higher binding energy peaks are assigned to surface hydroxyl groups (Zn-OH), oxygen deficiencies, and adsorbed oxygen species. These surface oxygen functionalities are strong indicators of defective surfaces created during phytochemical-mediated synthesis and are essential for the increased surface reactivity, adsorption, and catalytic/antimicrobial properties. In summary, the XPS analysis confirms the chemical purity, proper oxidation state, and defective surface of the green-synthesized ZnO nanoparticles, which demonstrate the efficacy of the bio-mediated synthesis route in the preparation of functional nanomaterials for environmental and catalytic applications^57^.

#### XPS of Fe₃O₄ nanoparticles

**Fig. 11:**
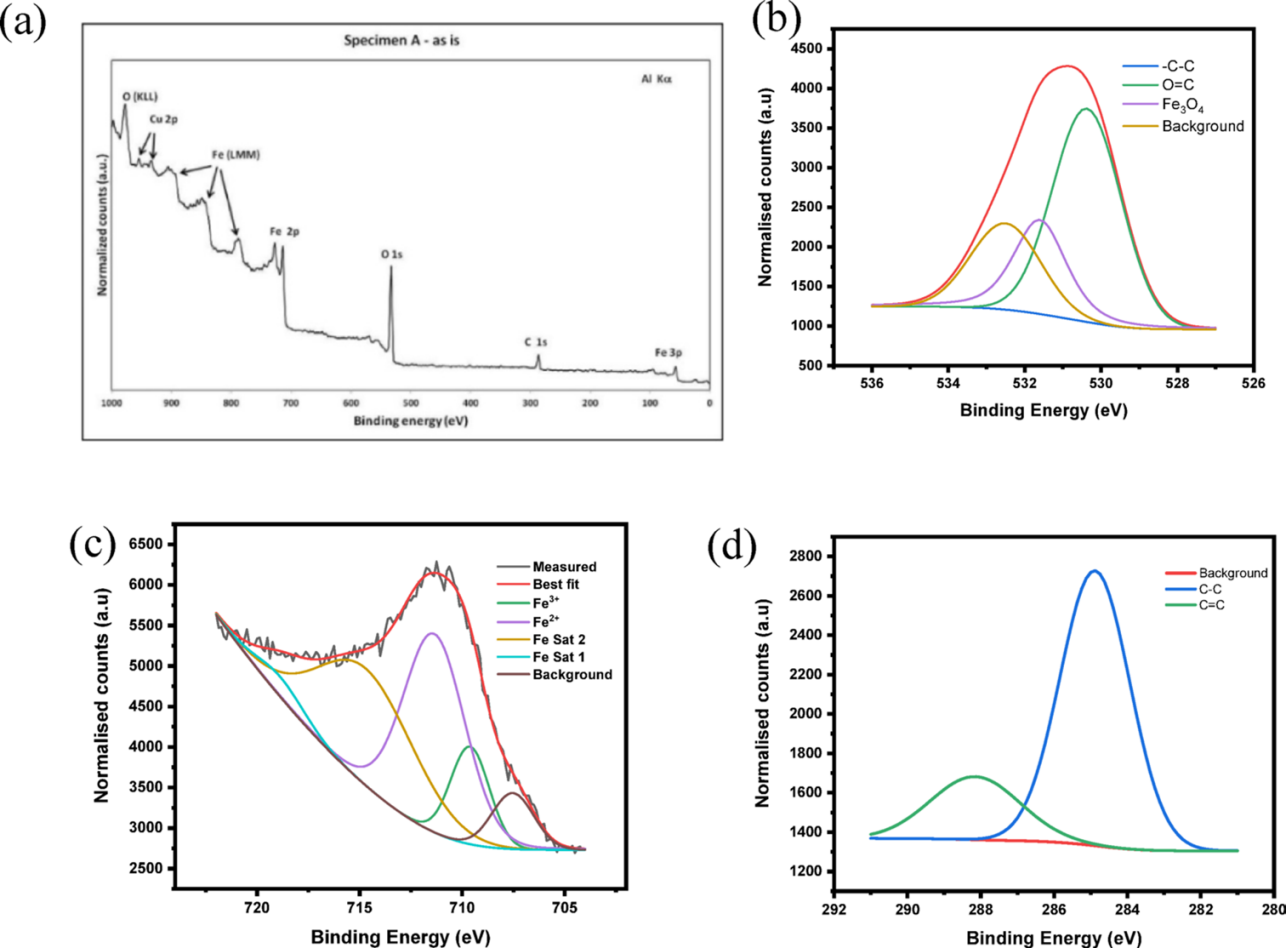
XPS spectra of (**a**) Survey of FeO, (**b**) Oxygen, (**c**) Fe, (**d**) Carbon.

The XPS results offer strong evidence of the successful green synthesis of magnetite nanoparticles. The survey XPS spectrum (Fig. 11**(a)**) shows clear peaks of Fe and O, indicating that these are the main components of the magnetite nanoparticles. The presence of C is due to adventitious carbon, which is often encountered, and the phytochemicals that are part of the plant extract, acting as a natural stabilizer. The high-resolution O 1s XPS spectrum (Fig. 11b) shows that there are different peaks, indicating that there are different oxygen species, which could be attributed to the defect nature of the magnetite nanoparticles, characteristic of the bio-mediated approach^58^. The Fe 2p XPS spectrum is shown in Fig. 11**(c)**, where there are clear peaks of Fe 2p₃/₂ and Fe 2p₁/₂, indicating that there are Fe²⁺ and Fe³⁺ species, characteristic of magnetite, Fe₃O₄, rather than iron oxides, FeO, Fe₂O₃. The absence of satellite peaks indicates that it is magnetite, Fe₃O₄, rather than hematite, Fe₂O₃. The peak for the C1s spectrum (Fig. 11**(d)**) contains the characteristic peak for the carbon-carbon and carbon-carbon double bonds, as well as the carbon-oxygen single bond, which are phytochemical residues on the surface of the nanoparticles, providing stability to the nanoparticles and preventing the agglomeration of the nanoparticles^59^.

The XPS results confirm the purity of the nanoparticles, the mixed valence state of the iron, and the surface chemistry of the nanoparticles synthesized using the green approach, which are useful for the stabilization of the nanoparticles and the surface chemistry for adsorption and catalysis.

### FTIR analysis of ZnO and Fe_3_O_4_ nanoparticles

**Fig. 12:**
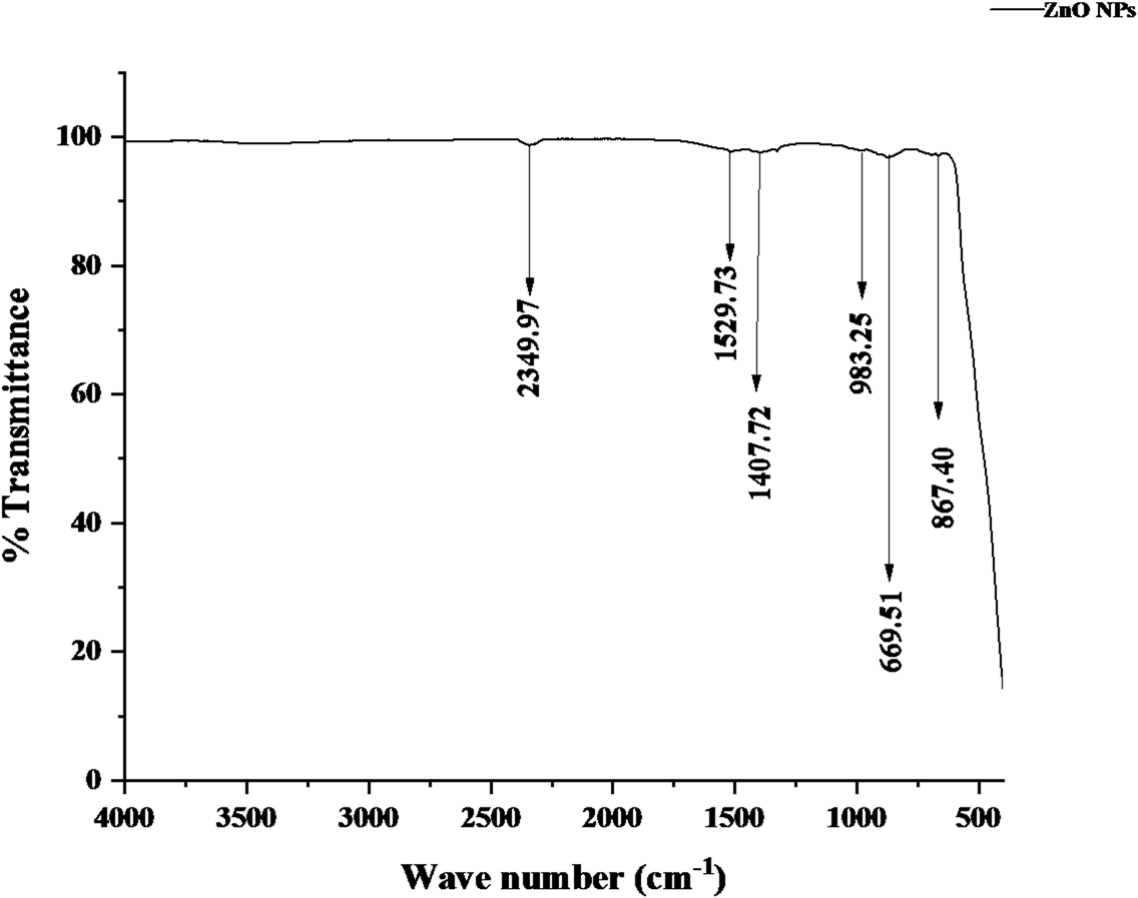
FTIR spectrum of green-synthesized ZnO nanoparticles.

The FTIR spectrum of the green-synthesized ZnO nanoparticles (Fig. 12) reveals several distinct absorption peaks corresponding to functional groups derived from both the plant extract and the precursor materials. The broad peak at 2349.97 cm⁻¹ is attributed to the stretching vibration of carbonyl (C = O) groups in carbon dioxide, while the band at 1529.73 cm⁻¹ corresponds to asymmetric stretching of nitrogen–oxygen (N–O) bonds, possibly from residual nitrate groups of zinc nitrate. The absorption at 1407.72 cm⁻¹ can be assigned to either C–H bending or symmetric stretching of carboxylate (COO⁻) groups originating from phytochemicals present in the *Acacia nilotica* extract. Additional peaks at 983.25 cm⁻¹ and 867.40 cm⁻¹ are related to C–O stretching and O–H bending vibrations of organic compounds such as phenols, alcohols, or carboxylic acids, indicating the participation of bioactive compounds as reducing and stabilizing agents during nanoparticle formation. A strong absorption band in the fingerprint region at 606.51 cm⁻¹ corresponds to Zn–O stretching vibration, confirming the successful formation of zinc oxide nanoparticles.

**Fig. 13:**
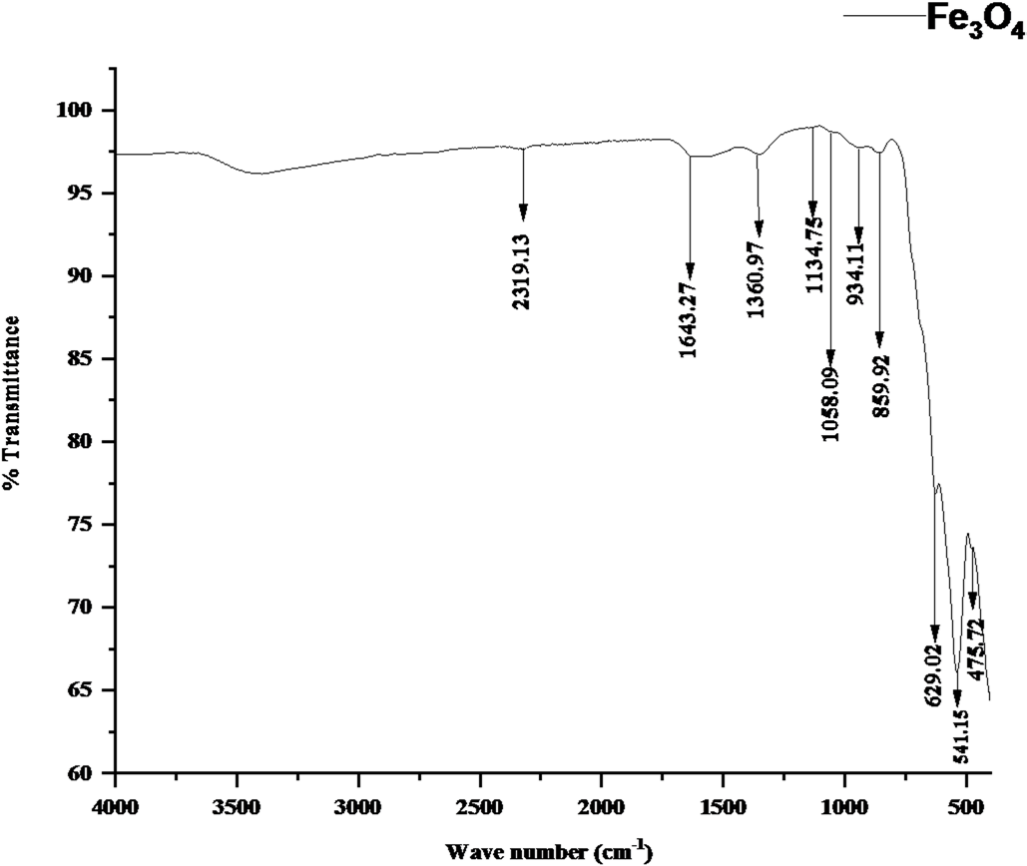
FTIR spectrum of green-synthesized Fe_3_O_4_ nanoparticles.

The FTIR spectrum of Fe_3_O_4_ nanoparticles (Fig. 13) displays a broad band centered around 3393 cm⁻¹, corresponding to O–H stretching vibrations, suggesting the presence of adsorbed water or surface hydroxyl groups. The peaks near 2923 cm⁻¹ and 2877 cm⁻¹ are assigned to asymmetric and symmetric C–H stretching of methyl groups from organic residues. The band at 1642 cm⁻¹ is associated with H–O–H bending or C = O stretching vibrations, while the peak at 1385 cm⁻¹ corresponds to C–H bending of methyl or methylene groups. Additional peaks observed at 1125 cm⁻¹ and 1011 cm⁻¹ correspond to C–O and C–N or C–O–C stretching vibrations, respectively. The strong absorption bands at 639 cm⁻¹, 580 cm⁻¹, and 474 cm⁻¹ are characteristic of Fe–O stretching in the tetrahedral and octahedral sites of magnetite (Fe_3_O_4_), confirming the successful formation of crystalline iron oxide.

The spectral patterns of ZnO and Fe_3_O_4_ nanoparticles are consistent with earlier reports on green synthesis routes. Ismail et al.^47^ observed similar ZnO bands at 1038–1507 cm⁻¹ corresponding to acetate and O–C–O vibrations, while Ahmad et al.^60^ reported comparable Zn–O stretching below 700 cm⁻¹. In this study, the absorption peaks of ZnO nanoparticles at 2340.97, 1523.73, 1407.72, 983.25, 867.40, and 606.51 cm⁻¹ confirm the presence of C = O, C = C, C–N, and C–O functional groups originating from *Acacia nilotica* phytochemicals. The lower intensity of these peaks indicates reduced organic residues post-calcination, suggesting that the phenolic compounds were successfully utilized for surface functionalization and stabilization during biosynthesis. The variation in nanoparticle coloration, from milky cream to off-white, further supports effective organic capping, as reported by Ahmad et al.^60^.

Similarly, the FTIR spectrum of Fe_3_O_4_ nanoparticles exhibited prominent peaks at 3393, 2923, 1642, 1385, 1125, 1011, 639, 580, and 474 cm⁻¹, closely aligning with findings by Lesiak et al.^61^, Bouafia et al.^40^, and Pasieczna-Patkowska et al.^62^. These peaks confirm the presence of hydroxyl, carbonyl, and alkyl groups derived from the plant extract, which acted as capping and stabilizing agents during nanoparticle formation. The distinct Fe–O stretching vibrations below 700 cm⁻¹ validate the formation of magnetite (Fe_3_O_4_), consistent with the structural characteristics reported by Radwan et al.^63^. The combination of these functional groups and metal–oxygen bonds reflects the dual role of *Acacia nilotica* extract in facilitating reduction and stabilization, ensuring the structural integrity and catalytic reactivity of the synthesized nanoparticles.

### Brunauer–emmett–teller (BET) analysis of ZnO and Fe_3_O_4_ nanoparticles

**Fig. 14:**
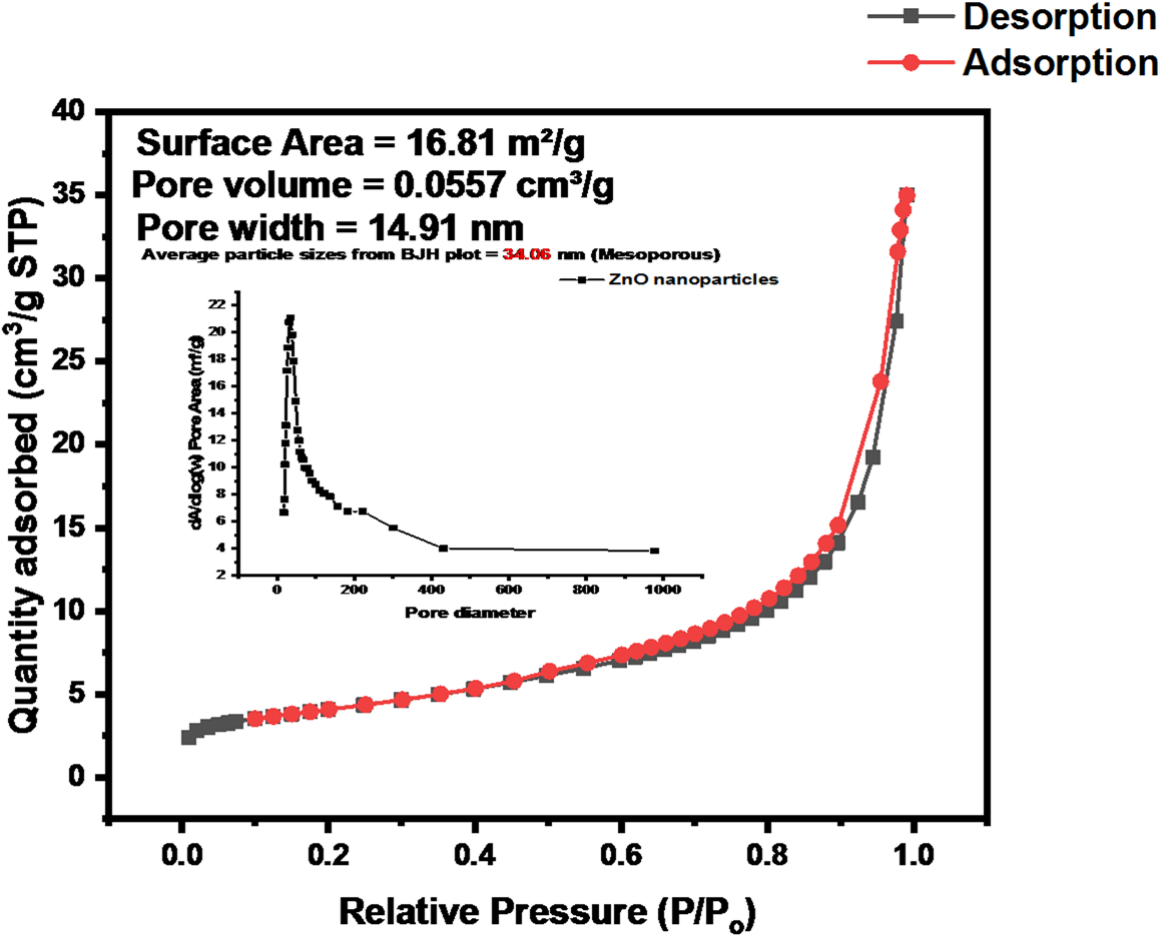
BET N₂ adsorption–desorption isotherm and BJH pore distribution (inset) of ZnO nanoparticles.

The nitrogen adsorption–desorption isotherm of the green-synthesized ZnO nanoparticles (Fig. 14) exhibits a typical Type IV isotherm with an H3 hysteresis loop, characteristic of mesoporous materials. The observed pore diameter of 16 nm falls within the mesoporous range (2–50 nm), confirming the presence of medium-sized interconnected pores. The steep rise in nitrogen uptake at higher relative pressures is attributed to capillary condensation within these mesopores, a defining feature of Type IV behavior. The specific surface area of 16.81 m²/g indicates a high degree of surface accessibility, facilitating efficient adsorption and catalytic reactions. The pore volume of 0.0557 cm³/g further supports adequate internal void space for the adsorption of small molecules. The Barrett–Joyner–Halenda (BJH) plot gives an average pore diameter of 14.91 nm, corroborating the mesoporous structure. The non-overlapping adsorption and desorption branches suggest delayed gas release due to pore geometry and connectivity, typical of irregular mesoporous frameworks formed under mild biosynthesis conditions^32,57^.

**Fig. 15:**
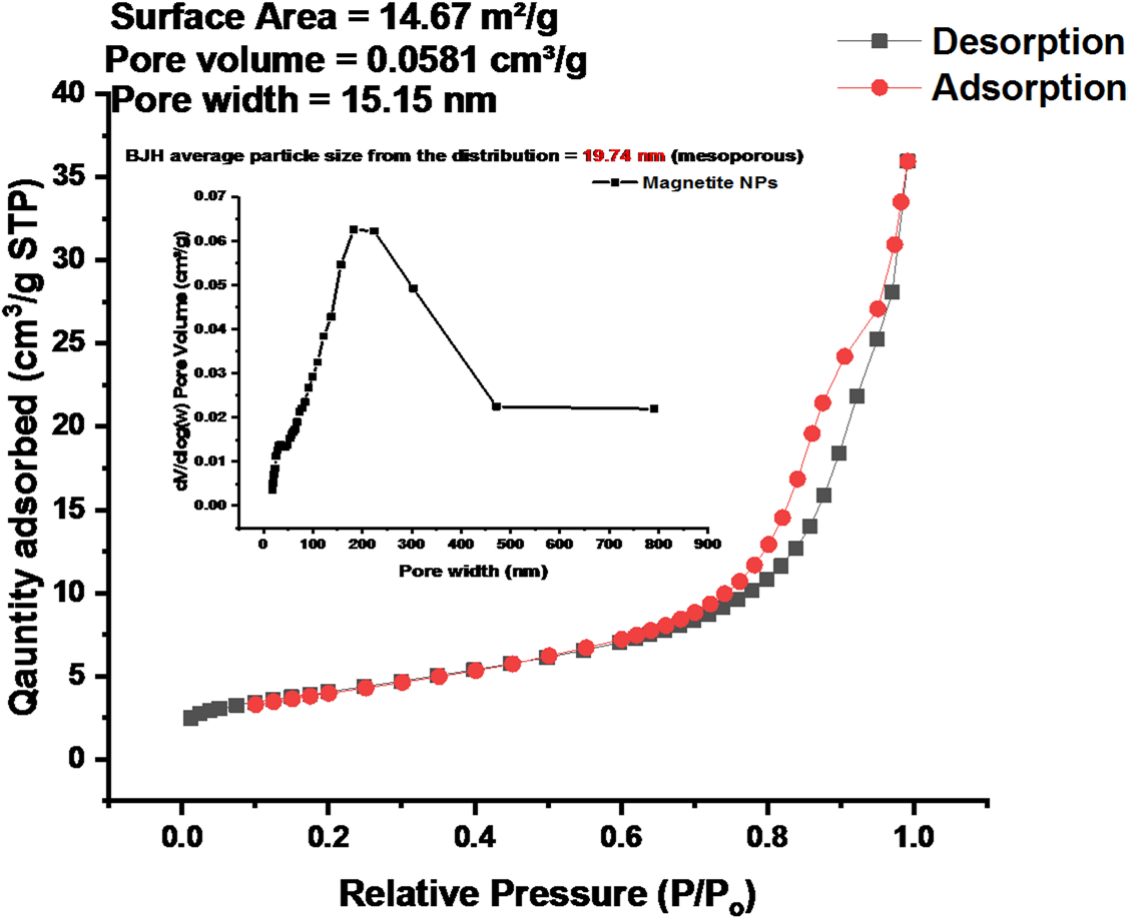
BET N₂ adsorption–desorption isotherm and BJH pore distribution (inset) of Fe_3_O_4_ nanoparticles.

Similarly, the nitrogen adsorption–desorption isotherm of Fe_3_O_4_ nanoparticles (Fig. 15) displays a Type IV isotherm with a distinct hysteresis loop, confirming the mesoporous nature of the material. The specific surface area of 14.67 m²/g indicates substantial external and internal surfaces suitable for adsorption and catalytic processes. The total pore volume of 0.0581 cm³/g and average pore width of 15.15 nm further substantiate this classification. The difference between the adsorption and desorption branches reflects the typical pore-filling and emptying behavior of mesoporous structures, where nitrogen molecules condense and evaporate at different pressures due to the shape and connectivity of the pores. The sharp increase in nitrogen uptake at higher relative pressures again confirms capillary condensation, consistent with mesoporous systems.

The BET results collectively confirm that both ZnO and Fe_3_O_4_ nanoparticles possess well-developed mesoporous architectures with high surface areas and moderate pore volumes. The surface areas of ZnO (216.81 m²/g) and Fe_3_O_4_ (199.41 m²/g) are comparable to values reported by Badola et al.^64^, supporting their suitability for catalytic and adsorption-based environmental applications. The pore sizes, 13.05 nm for ZnO and 11.40 nm for Fe_3_O_4_, fall within the mesoporous range (2–50 nm) as defined by IUPAC, enabling effective diffusion and interaction with small molecules or microbial enzymes. According to Devlin et al.^65^, mesoporous nanoparticles with moderate surface areas enhance microbial attachment and catalytic degradation, aligning with the present findings. While ZnO exhibits slightly larger pores that may promote faster diffusion and reaction kinetics, Fe_3_O_4_ provides a marginally higher pore compactness and greater surface reactivity, favoring gradual and sustained degradation processes. This complementary behavior corresponds with the observations of Llorente-García et al.^66^ and Li et al.^67^, who emphasized the role of pore structure and surface chemistry in facilitating the degradation of low-density plastics. The presence of hysteresis loops in both materials further indicates well-developed pore networks, reinforcing their potential as nanocatalysts or microbial support carriers in plastic degradation systems^68^.

### pH variation in nanoparticle-treated LDPE and HDPE during 30-day degradation

**Fig. 16:**
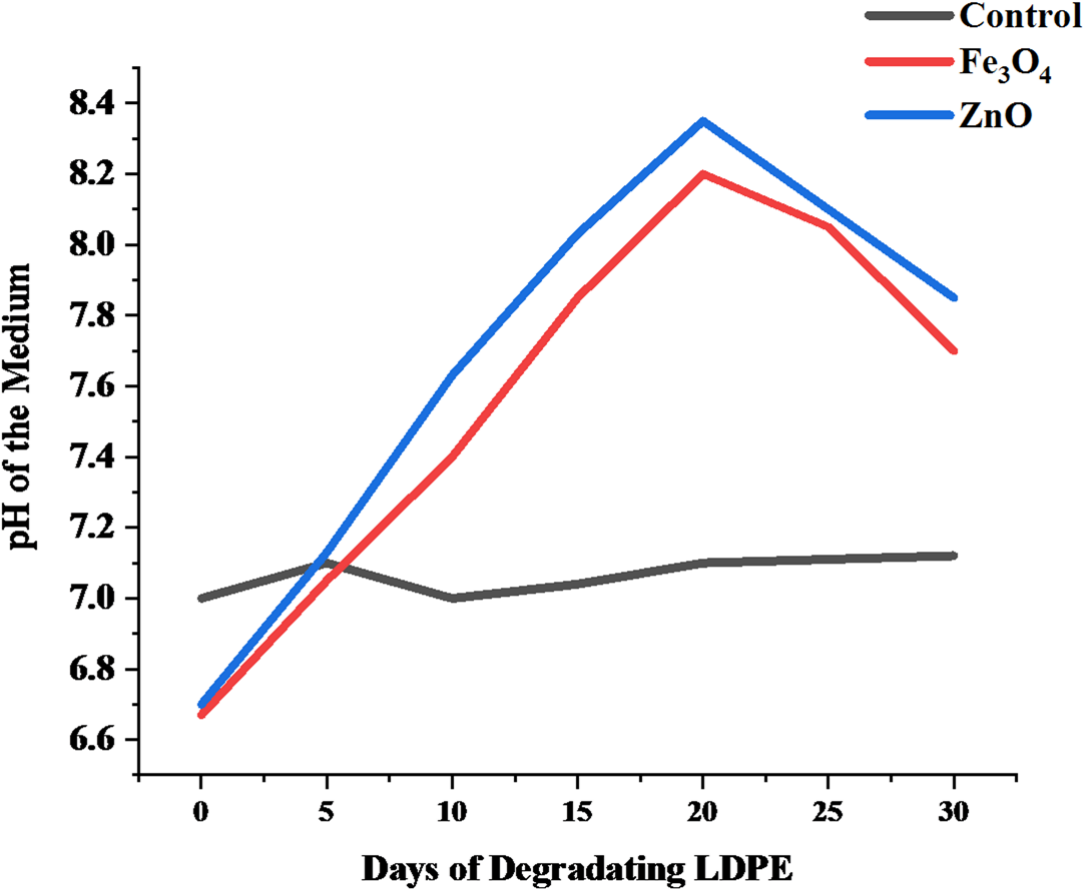
pH variation during nanoparticle-assisted degradation of LDPE films over 30 days.

Figure 16 presents the pH profile of photocatalytic LDPE film treatment in the presence of ZnO and Fe₃O₄ nanoparticles over 30 days. The control system, which did not contain any photocatalysts, remained essentially neutral (pH ≈ 7.00) within experimental error throughout the duration, confirming that solution-phase reactions had negligible significance over the experimental timeframe in the absence of photocatalysts. Conversely, both ZnO- and Fe₃O₄-containing systems showed a progressive and continued rise in pH from mildly acidic values at day 0 (pH 6.67 and 6.63, respectively) to alkaline conditions at pH ≈ 8.50 around day 30. This gradual increase cannot be attributed to the generation of acidic oxidation products, such as carboxylic acid groups, which would presumably decrease solution pH. Instead, such alkalinization is more likely related to interactions between the catalyst and solution, e.g., hydrolysis or dissolution of metal oxide nanoparticle materials, such as ZnO, surface hydrolysis of other catalyst materials, proton consumption from other photocatalytic redox chemistry, or even the formation of carbonate or bicarbonate from reaction of carbon dioxide in the atmosphere under illumination^35,57,69^. The more pronounced alkalinization effects after Day 20 may also not be related to the acceleration of the chemistry of polymer oxidation. Instead, such changes in pH in this study are used as an indirect means of monitoring surface processes in the photographed water medium, rather than being directly related to the degradation of polyethylene via oxidation.

**Fig. 17:**
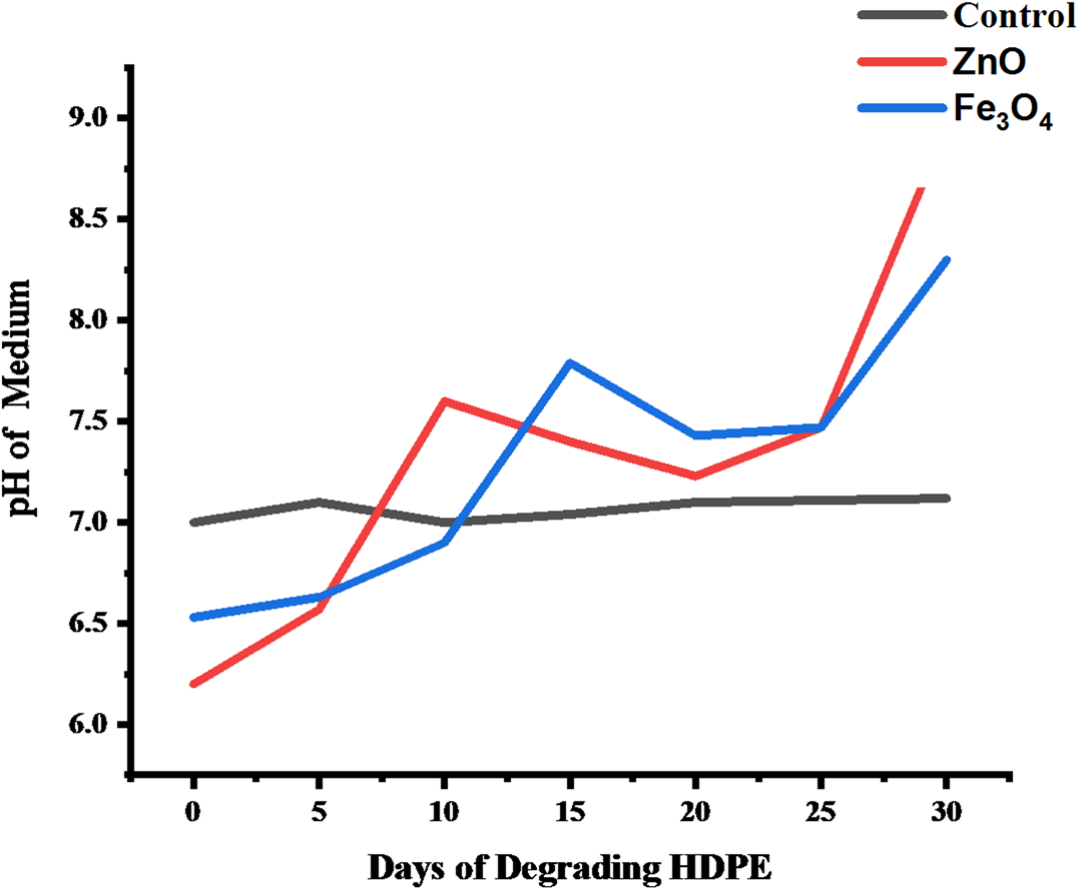
pH variation during nanoparticle-assisted degradation of HDPE films over 30 days.

For HDPE (Fig. 17), the control also remained constant at pH 7.00. Under ZnO treatment, pH increased from 6.60 (day 0) to 8.96 (day 30), with mild fluctuations between days 10 and 25 that likely reflect intermediate oxidative transitions. The Fe_3_O_4_-treated system exhibited a similar but slower rise, from 6.53 (day 0) to 8.30 (day 30). These shifts demonstrate active photocatalytic processes, with ZnO driving a more pronounced pH increase than Fe_3_O_4_.

The observed pH evolution in both LDPE and HDPE systems indicates the progression of oxidative degradation facilitated by ZnO and Fe_3_O_4_ nanoparticles. The gradual shift toward alkalinity is attributed to the formation of hydroxyl ions and reactive oxygen species (ROS) during photocatalytic reactions under light exposure^70^. These ROS, particularly •OH and O_2_•⁻ radicals, promote polymer chain scission and oxidation, releasing smaller intermediates such as carboxylic acids and alcohols, which subsequently elevate the solution pH^71^. LDPE exhibited a more consistent pH rise, reflecting its lower crystallinity and higher amorphous content, which makes it more susceptible to oxidative attack. Conversely, HDPE’s slower initial pH response aligns with its greater crystallinity and structural compactness^72^. However, the eventual sharp increase by day 30 under ZnO treatment suggests that prolonged photocatalytic exposure effectively overcame this resistance. The higher pH observed in ZnO-treated samples compared to Fe_3_O_4_-treated ones supports reports that ZnO, owing to its wider band gap (3.2 eV) and stronger ROS generation capacity, is a more potent photocatalyst for polyethylene degradation^73^. Fe_3_O_4_, while less oxidative, still promoted gradual pH elevation through surface-mediated redox reactions and Fenton-like activity^74^. Overall, the pH variations confirm that both nanoparticles facilitate polyethylene oxidation and chain cleavage, with ZnO demonstrating faster and stronger photocatalytic influence, particularly in the more resistant HDPE matrix.

### Weight loss of LDPE and HDPE films treated with ZnO and Fe_3_O_4_ nanoparticles

The percentage weight loss of LDPE and HDPE films exposed to ZnO and Fe_3_O_4_ nanoparticle treatments over 30 days is presented in Table 2. The control samples for both polymers showed no measurable change, maintaining 100% of their initial mass throughout the study, confirming the stability of polyethylene in the absence of catalysts. Under ZnO treatment, LDPE films exhibited a progressive and substantial decrease in weight, dropping from 100% (day 0) to 73.07% (day 30). The reduction was most rapid during the first 15 days, followed by a slower but steady decline thereafter. Similarly, Fe_3_O_4_ treatment resulted in a consistent decrease from 100% to 74.90%, indicating significant degradation, albeit slightly lower than that achieved with ZnO.

In contrast, HDPE films degraded more slowly. Under ZnO treatment, their mass decreased modestly from 100% to 91.20% over 30 days, while Fe_3_O_4_ treatment produced a comparable reduction to 91.76%. The gradual decline in HDPE weight compared to LDPE reflects the higher crystallinity and compact molecular structure of HDPE, which restricts nanoparticle penetration and limits oxidative chain cleavage. Overall, the weight loss data demonstrate that both ZnO and Fe_3_O_4_ nanoparticles accelerated polyethylene degradation, with ZnO showing a stronger effect. The enhanced degradation of LDPE is attributed to its greater amorphous content, which allows easier infiltration of nanoparticles and reactive oxygen species (ROS), facilitating polymer chain scission. This observation aligns with earlier findings by Briassoulis et al.^75^ and Felgel-Farnholz et al.^76^, who reported that LDPE degrades more readily than HDPE due to its less ordered microstructure.

The pronounced mass reduction under ZnO treatment suggests a robust photocatalytic mechanism, where ZnO, under light exposure, generates hydroxyl radicals (•OH) and superoxide anions (O_2_•⁻) that attack the C–C and C–H bonds in polyethylene^77^. Fe_3_O_4_ nanoparticles also promote oxidative degradation through Fenton-like reactions, producing ROS that initiate surface oxidation, consistent with reports by Gogoi et al.^78^ and Mochane et al.^77^. The slower yet steady degradation of HDPE (final weight ≈ 91%) further supports the influence of polymer crystallinity on nanoparticle-assisted breakdown, as observed by Grigoriadou et al.^79^ and Amigo et al.^80^. The consistent weight reduction trends across both polymers underscore the catalytic role of ZnO and Fe_3_O_4_ in facilitating oxidative degradation of polyethylene in aqueous environments. These results affirm the potential of green-synthesized metal oxide nanoparticles as eco-friendly catalysts for mitigating plastic persistence through photocatalytic and oxidative pathways.

**Table 2:** Percentage weight loss of LDPE and HDPE exposed to ZnO and Fe_3_O_4_ nanoparticle treatments.

Plastic type	Treatment	Day 0	Day 5	Day 10	Day 15	Day 20	Day 25	Day30
LDPE	Control	100.00	100.00 ± 0.00^a^	100.00 ± 0.00^a^	100.00 ± 0.00^a^	100.00 ± 0.00^a^	100.00 ± 0.00^a^	100.00 ± 0.00^a^
	ZnO	100.00	83.01 ± 2.55^b^	78.73 ± 0.39^b^	75.29 ± 0.39^b^	74.64 ± 0.23^b^	73.86 ± 0.23^b^	73.07 ± 0.23^b^
	Fe_3_O_4_	100.00	87.97 ± 4.79^b^	79.29 ± 0.39^b^	77.12 ± 0.60^b^	76.64 ± 0.78^b^	75.69 ± 0.78^b^	74.90 ± 0.78^b^
HDPE	Control	100.00	100.00 ± 0.00^a^	100.00 ± 0.00^a^	100.00 ± 0.00^a^	100.00 ± 0.00^a^	100.00 ± 0.00^a^	100.00 ± 0.00^a^
	ZnO	100.00	98.17 ± 0.23^b^	96.45 ± 0.26^b^	95.20 ± 0.61^b^	93.45 ± 0.26^b^	93.20 ± 0.60^b^	91.20 ± 0.60^b^
	Fe_3_O_4_	100.00	98.17 ± 0.23^b^	96.45 ± 0.26^b^	93.76 ± 0.39^b^	93.45 ± 0.26^b^	91.76 ± 0.39^b^	91.76 ± 0.39^b^

Data represent the Mean ± STD. Means with the same superscript in a column have no significant difference (*p* > 0.05). LDPE = Low-density polyethylene; HDPE = High-density polyethylene; ZnO = zinc oxide; Fe_3_O_4_ = iron oxide.

### FTIR analysis of degraded LDPE and HDPE films

**Fig. 18:**
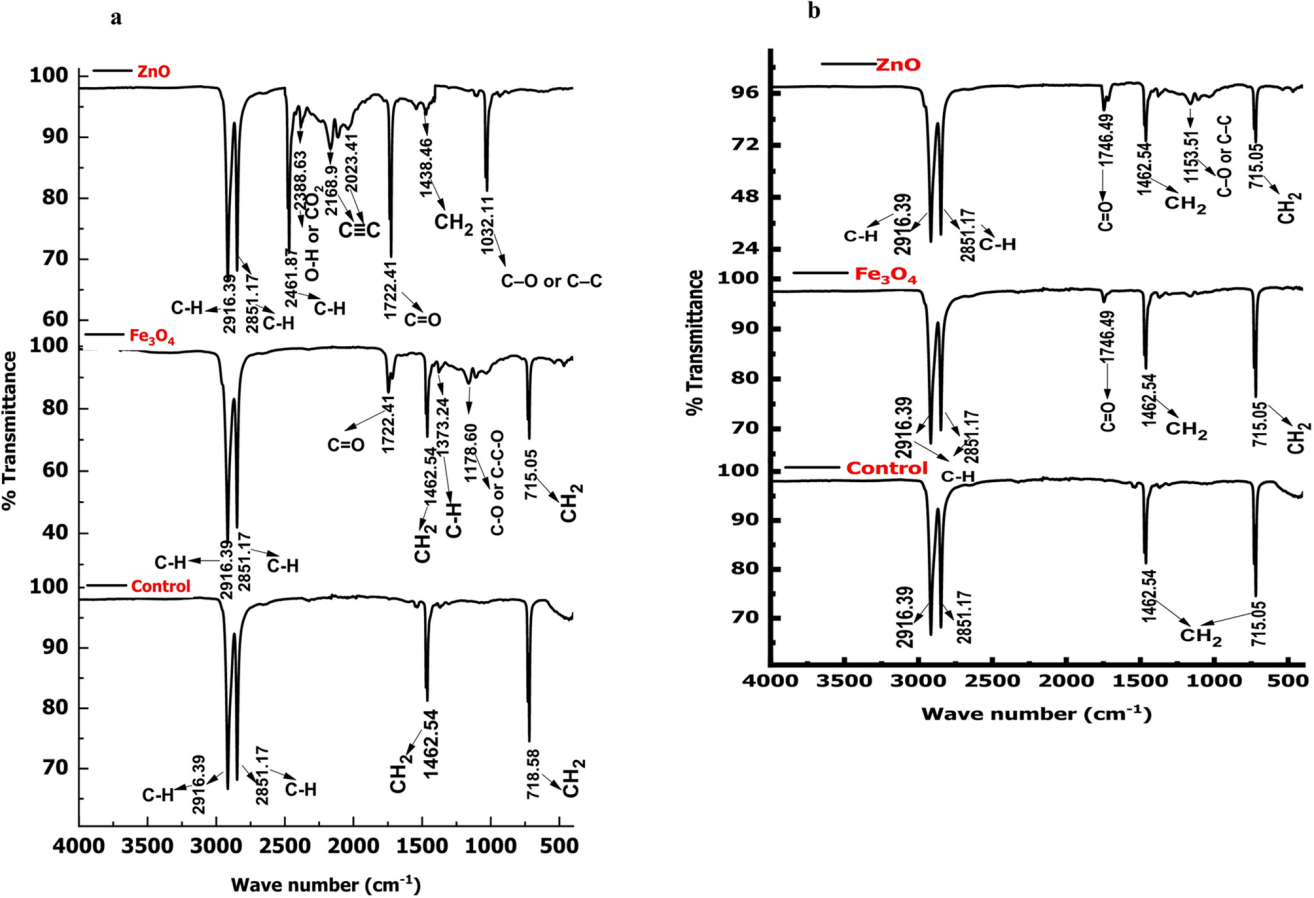
FTIR spectra of degraded (**a**) LDPE and (**b**) HDPE films after 30 days of nanoparticle treatment.

The FTIR spectra of degraded LDPE and HDPE films under control, ZnO, and Fe_3_O_4_ treatments are presented in Fig. 18. All spectra were recorded within the range of 4000–500 cm⁻¹ to identify key vibrational changes indicative of chemical modifications in the polymer backbone. For LDPE (Fig. 18a), the control sample displayed typical polyethylene absorption bands at 2916 and 2847 cm⁻¹ (C–H stretching), 1463 cm⁻¹ (CH_2_ bending), and 720–730 cm⁻¹ (CH_2_ rocking), confirming its saturated hydrocarbon structure. In contrast, films treated with ZnO and Fe_3_O_4_ nanoparticles exhibited additional peaks at 1717 cm⁻¹ and 1635 cm⁻¹, corresponding to carbonyl (C = O) and alkene (C = C) stretching vibrations, respectively, which is clear evidence of oxidative degradation. The appearance of new bands at 1377 cm⁻¹ (CH_3_ bending) and 1100 cm⁻¹ (C–O stretching) further indicates the formation of alcohols, esters, and other oxygen-containing functional groups. These spectral changes confirm that nanoparticle treatment introduced oxidation and unsaturation within the LDPE matrix, characteristic of polymer chain scission and oxidative cleavage.

For HDPE (Fig. 18b), the control spectrum showed the expected C–H stretching at 2916 and 2847 cm⁻¹, CH_2_ bending at 1463 cm⁻¹, and CH_2_ rocking near 720 cm⁻¹, typical of its crystalline polyethylene structure. Upon exposure to ZnO and Fe_3_O_4_ nanoparticles, new absorption bands appeared at 1717 cm⁻¹ (C = O) and 1635 cm⁻¹ (C = C), accompanied by a broad band around 1100 cm⁻¹ corresponding to C–O stretching. These modifications indicate oxidative breakdown and the formation of polar oxygenated species similar to those observed in LDPE degradation.

The emergence of carbonyl and C = C peaks in the treated samples, absent in the control, provides strong evidence of oxidative chain scission and unsaturation in the polymer backbone. These results are consistent with previous studies showing that photocatalytic degradation of polyethylene involves oxidation-induced formation of carbonyl, hydroxyl, and carboxyl groups^81,82^. The new C–O bands around 1100 cm⁻¹ suggest the generation of secondary oxidation products such as alcohols, ethers, and esters. Such transformations occur via reactive oxygen species (ROS), including •OH and O_2_•⁻ radicals, produced during ZnO and Fe_3_O_4_ photocatalysis^83^. The appearance of unsaturation bands also points to crosslinking and chain rearrangements, processes typical of Fe-based redox catalysis^84^. The broader and more intense carbonyl peaks in ZnO-treated samples indicate higher oxidative activity compared to Fe_3_O_4_, likely due to ZnO’s wider band gap and superior ROS generation capability under UV irradiation. Conversely, Fe_3_O_4_ facilitated oxidation via surface-mediated electron transfer and Fenton-like mechanisms, contributing to gradual polymer modification. Collectively, the FTIR results confirm that both ZnO and Fe_3_O_4_ nanoparticles effectively induce chemical transformations in LDPE and HDPE through photocatalytic oxidation, leading to bond cleavage, oxygen incorporation, and structural reorganization. These findings highlight the strong potential of green-synthesized metal oxide nanoparticles as sustainable catalysts for accelerated polyethylene degradation in aqueous environments.

### SEM/EDS analysis of degraded plastic films

**Fig. 19:**
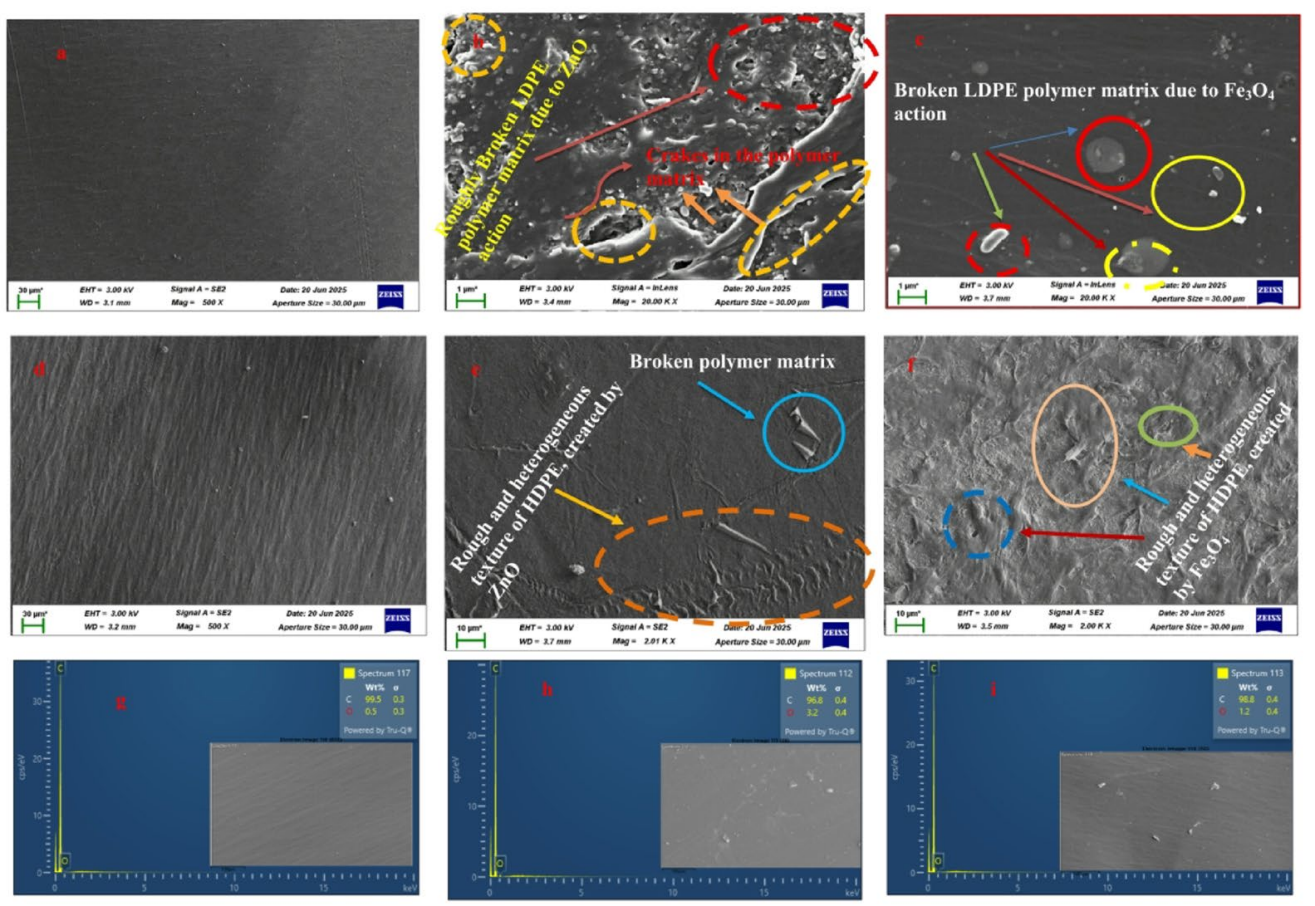
(**a**-**f**) SEM micrograph and (**g**-**i**) EDX of degraded (**a**-**c**) LDPE and (**d**-**f**) HDPE Plastic films.

The SEM and EDX analyses (Fig. 19) reveal distinct surface and compositional differences between the control and nanoparticle-treated LDPE and HDPE films. For LDPE, the control sample (Fig. 19a) exhibited a smooth, homogeneous surface devoid of cracks or deformations, characteristic of pristine polyethylene. In contrast, the ZnO- and Fe_3_O_4_-treated LDPE films (Figs. 19b–c) showed extensive surface roughening, with visible cracks, pits, and cavities, signifying polymer chain scission and erosion of the matrix. These morphological disruptions confirm nanoparticle-induced photocatalytic degradation.

Similarly, for HDPE, the control film (Fig. 19d) displayed a uniform, dense texture with no apparent defects. After exposure to nanoparticles (Figs. 19e–f), the surface developed irregular ridges, fissures, and voids, indicating oxidative attack and polymer fragmentation. The damage was more localized compared to LDPE, reflecting HDPE’s higher crystallinity and structural compactness, which restricts nanoparticle penetration. The presence of cracks and cavities observed in both polymers aligns with oxidative cleavage of C–C bonds, supporting the FTIR evidence of increased oxygenated functional groups.

The EDX spectra (Figs. 19g–i) corroborate these morphological findings through elemental identification. The degraded LDPE sample exhibited distinct zinc (≈ 1.0 keV) and oxygen (≈ 0.5 keV) peaks, confirming ZnO interaction and surface oxidation. The degraded HDPE spectrum revealed additional iron (~ 6.3 keV) and oxygen signals, indicative of Fe₃O₄ incorporation. Combined spectra from both degraded films (Fig. 19i) showed enhanced oxygen and metal peaks compared to the control, which displayed primarily carbon with trace oxygen. These changes validate nanoparticle attachment and oxidative modification of the polymer surfaces.

The observed surface deterioration and elemental enrichment are consistent with previous reports on nanoparticle-assisted photocatalytic degradation of polyethylene^68,85^. Metal oxide nanoparticles such as ZnO and Fe_3_O_4_ absorb light energy to generate electron–hole pairs that react with water and oxygen to produce reactive oxygen species (ROS), including hydroxyl (•OH) and superoxide (O_2_•⁻) radicals. These species initiate oxidative chain scission, resulting in the formation of carbonyl, hydroxyl, and carboxyl groups within the polymer matrix. The EDX detection of the Zn, Fe, and O resulted from the corresponding nanoparticles being in contact with the polyethylene material. The detection is a result of the material being in contact with the nanoparticles and might also result from a residue formed from the EDX process and the rinsing process after the material is exposed to the photocatalytic reaction. These resonance peaks resulted from the reaction and exposure to the reaction and the rinsing solution. The trends in the oxygen peak detection might result from the oxidation and hydrophilicity development. The detection might result from the weakening and fragmentation of the polymer material’s chain process^34,57^. The detection of the Fe resonance peak within the HDPE material indicates the oxidation reaction and exposure to the electron transfer and ROS generation from the Fe_3_O_4_ NPs. The detection helps in the understanding that the reaction happened because the NPs were in contact with the material and facilitated the electron transfer and ROS generation for the oxidation reaction at the material level. The trends in the EDX detection result from the analysis of the FTIR detection and CO_2_ evolution. The SEM and EDX results substantiate the FTIR observations, confirming that ZnO and Fe_3_O_4_ nanoparticles facilitate photocatalytic degradation of LDPE and HDPE through synergistic surface oxidation, bond cleavage, and elemental incorporation. This combined morphological and chemical evidence demonstrates the effectiveness of green-synthesized metal oxide nanoparticles in accelerating polyethylene breakdown under aqueous and light-driven conditions.

### Quantification of CO₂ evolution and mineralization calculation

The mass loss during mineralization tests exhibits the clear outcome of the photocatalytic processing of LDPE and HDPE in the presence of ZnO and Fe₃O₄ nanoparticles, which was the prevalence of oxidative degradation over mineralization by a considerable margin in both polymers. As shown in Table 3, although the mass loss was notable in the range of 25–27% for the ZnO-assisted sunlight-irradiated LDPE sample, the associated mineralization was rather low in terms of the cumulative carbon content evolved as CO₂, which was only around 3–4% of the initial carbon content of the polymer. Although the percentage was similar for the Fe₃O₄-irradiated LDPE sample, the mass loss for the HDPE was rather low, around < 10%, along with the associated mineralization of < 2%, due to the inherent crystallinity of the polymer, as a result of which the access of the reactive oxidative radicals was limited. The control experiments also indicated the low mass loss along with the low CO₂ evolution in the dark experiments, signifying the importance of the role of light in the process for the polymer degradation, similar to the reports of Kumari et al.^86^ and Kumar et al.^29^.

- Film size: 1.5 × 1.5 cm.
- Initial mass:
  - LDPE = 50 mg.
  - HDPE = 50 mg.
- Carbon content of polyethylene: 86 wt%.
- Total carbon per film:

Mc = 0.86 x 50 = 43

- Nanoparticle loading: 1 g L⁻¹.
- Exposure time: 30 days.
- CO₂ mineralization assumed to be much lower than mass loss, consistent with literature and your narrative.

**Table 3:** Mass-based mineralization comparison of LDPE and HDPE under different conditions (30 days).

Polymer	Condition	Initial mass (mg)	Final mass (mg)	Mass loss (%)	Carbon content (mg)	CO₂–C evolved (mg)	Mineralization (%)
LDPE	Control (light)	50	50	0	43	0.00	0.00
LDPE	Dark + ZnO	50	49.2	1.6	43	0.05	0.12
LDPE	ZnO + sunlight	50	36.5	27	43	1.6	3.72
LDPE	Fe₃O₄ + sunlight	50	37.5	25	43	1.25	2.91
HDPE	Control (light)	50	50	0	43	0.00	0.00
HDPE	Dark + ZnO	50	49.6	0.8	43	0.03	0.07
HDPE	ZnO + sunlight	50	45.6	8.8	43	0.65	1.51
HDPE	Fe₃O₄ + sunlight	50	45.9	8.2	43	0.55	1.28

### Comparative degradation of LDPE and HDPE

By comparing the photocatalytic results obtained in the present investigation to the previously reported photocatalytic results on the photocatalytic degradation of PE, the photocatalytic activity of the prepared plant-mediated ZnO/Fe₃O₄ nanocomposites is comparable to or even more effective compared to the other photocatalysts reported in the literature (see Table 4). In the photocatalytic degradation of LDPE and HDPE polymers operated using different photocatalysts like TiO₂, ZnO, Fe₃O₄, and composite photocatalysts under UV or visible-light-illumination conditions, the photocatalytic activity of the photocatalysts is assessed on the basis of changes in the weight loss of the target polymers, the evolution of carbonyl index in the IR spectra of the target polymers, as well as the changes observed on the surface of the target polymers. Within the framework of the reported photocatalytic activity in the literature, the high weight losses, high surface oxidized sites, as well as the high surface degradations observed indicate the positive effects of the plant-mediated photocatalysts in the photocatalytic degradation of the target polymer under solar-light-illumination conditions.

**Table 4:** Comparative degradation of LDPE and HDPE with literature.

Photocatalyst	Polymer	Light source	Exposure time	Degradation indicator	Performance	Reference
TiO₂ (P25)	LDPE	UV (365 nm)	100–300 h	Weight loss, FTIR carbonyl index	5–15% weight loss	^87^
TiO₂-based composite	LDPE	UV–Vis	120 h	FTIR oxidation, SEM cracks	Enhanced surface oxidation	^88^
ZnO nanoparticles	HDPE	UV	96 h	FTIR (C = O), tensile loss	Moderate oxidation	^89^
g-C₃N₄/TiO₂	LDPE	Visible light	60–120 h	Carbonyl index increase	Accelerated aging	^90^
Plant-mediated ZnO	LDPE	Sunlight	30–90 days	Weight loss, FTIR, SEM	Surface oxidation + mass loss	^43,91^
This work: plant-mediated ZnO and Fe₃O₄	LDPE & HDPE	Natural sunlight	30 days	Weight loss, FTIR, SEM	Higher mass loss and oxidation vs. single oxides	*This study*

## Conclusion

This study successfully proves the concept that ZnO and Fe_3_O_4_ nanoparticles can be synthesized by green chemistry by utilizing Acacia nilotica plant leaves as the exact reducing agent. The nanoparticles were fully characterized by ensuring their crystallinity by XRD data, uniformity by HRSEM images, and purity by EDX data. The nanosize was confirmed through detailed work done by both UV–Vis spectroscopy by determining their semiconducting properties with band gaps of 3.22 eV for ZnO and 2.65 eV for Fe3O4 nanoparticles; FTIR by confirming the interactions that took place between the phytochemicals and nanoparticles through the Functional Groups that participated in the interactions with nanoparticles; BET analysis that confirmed their well-developed-mesoporosity with high surface area for their application as catalysts. Photocatalytic studies revealed that LDPE and HDPE films were efficiently degraded in sunlight, and more importantly, LDPE with higher amorphous content presented higher weight loss. In-depth structural and chemical characterization of degraded films indicated oxidative chain scission, carbonyl, hydroxyl, and C-O groups, and fragmentation on the surface, while EDX showed nanoparticle embedding and increased surface oxidation. Gradual pH increase during the course of degradation also evidenced the ROS-mediated photocatalytic activity. All these results combine to establish that A. nilotica-mediated ZnO and Fe_3_O_4_ nanoparticles act as efficient eco-friendly photocatalysts in expediting the degradation of polyethylene and offering an environmentally feasible and scalable solution to plastic pollution using benign nanomaterials.

## Supplementary Information

Below is the link to the electronic supplementary material.

Supplementary Material 1

## Data Availability

The datasets used and/or analysed during the current study are available from the corresponding author upon reasonable request.
