# Supplementary material for: Photocatalytic degradation of polyethylene films using green-synthesized ZnO and Fe3O4 nanoparticles from Acacia nilotica
Source: Sci Rep. 2026 Mar 19;16:14212. doi: 10.1038/s41598-026-43013-w (PMC13139506; doi:10.1038/s41598-026-43013-w)
Supplement: Supplementary file 1 — Supplementary Material 1 [file 41598_2026_43013_MOESM1_ESM.docx]

**2.3S1. Phytochemical Screening**

Phytochemical screening was conducted following the method described by Ayyanar et al. [18] to identify and quantify key secondary metabolites such as saponins, alkaloids, flavonoids, tannins, and total phenols present in the powdered leaves of *Calotropis gigantea* (milkweed), *Azadirachta indica* (neem), *Mangifera indica* (mango), and *Acacia nilotica* (L.) (acacia).

**2.3S2. Determination of Total Phenolic Content**

The total phenolic content (TPC) was determined using the Folin–Ciocalteu colorimetric method as described by Lamuela‐Raventós [19]. Briefly, aliquots of the leaf extract were mixed with a small volume of Folin–Ciocalteu reagent, followed by the addition of sodium carbonate solution. The resulting mixture was incubated in the dark for 2 h to allow color development. Absorbance was measured at 765 nm using a UV–Vis spectrophotometer (Shimadzu UV-1800). Gallic acid was used as the calibration standard, and results were expressed as milligrams of gallic acid equivalents per gram of dry sample (mg GAE/g). All analyses were performed in triplicate to ensure accuracy.

**2.3S3. Determination of Tannin Content**

Tannin content was quantified using the method of Broadhurst and Jones [20] with slight modification. Powdered *A. nilotica* leaves were soaked in 70% aqueous acetone for 40 min and centrifuged at 10,000 rpm for 15 min. The supernatant was collected for analysis. A vanillin–HCl reagent was prepared by dissolving 0.5% vanillin in methanol, followed by the addition of 4% concentrated hydrochloric acid to form a vanillin–tannin complex. Equal volumes of the leaf extract and the reagent were mixed and incubated at 30°C for 20 min. The absorbance was recorded at 500 nm using a UV–Vis spectrophotometer. All measurements were performed in triplicate, and results were expressed as milligrams of catechin equivalents per gram of dry sample (mg CE/g).

**2.3S4 Determination of Flavonoid Content**

The total flavonoid content (TFC) was determined using the aluminum chloride colorimetric method described by Zhishen et al. [21], with modifications. Briefly, aliquots of the leaf extract were sequentially mixed with 5% (w/v) sodium nitrite, 10% (w/v) aluminum chloride, and 1 M sodium hydroxide. After 15 min of incubation at ambient temperature, absorbance was measured at 510 nm using a UV–Vis spectrophotometer. Quercetin was used as the standard (0–100 µg/mL), and results were expressed as milligrams of quercetin equivalents per gram of dry sample (mg QE/g). All experiments were conducted in triplicate to ensure reproducibility.

**2.3S5 Determination of Alkaloid Content**

The alkaloid content was quantified following the spectrophotometric procedure of Fazel et al. [22], with modifications. One gram of finely powdered *A. nilotica* leaf was extracted with 10 mL of a methanol–water solution (70:30 v/v) under reflux at 60°C for 2 h. The mixture was centrifuged at 3,000 rpm for 15 min, and the supernatant was filtered. For alkaloid quantification, 2 mL of the filtrate was reacted with 2 mL of Dragendorff’s reagent to form an orange-red precipitate. The precipitate was dissolved in 2 mL of 2 M hydrochloric acid, and absorbance was measured at 470 nm using a UV–Vis spectrophotometer (Shimadzu UV-1800). Caffeine (0–100 µg/mL) was used as the calibration standard. The calibration curve followed the equation *y = 0.012x + 0.004* (R² = 0.998). Alkaloid concentration was calculated using Equation (1):

$\text{Alkaloid content (mg/g)}=\frac{\text{C (µg/mL) × V (mL)}}{\text{W (g)}}$ (1)

where C = concentration obtained from the calibration curve, V = extract volume, and W = sample weight. Results were expressed as milligrams of caffeine equivalents per gram of dry sample (mg CE/g). Each test was performed in triplicate to ensure accuracy.

**2.3S6 Determination of Saponin Contentaminu**

Saponin content was determined using the method of Obadoni and Ochuko [23] and Edeoga et al. [24], with slight modifications. One gram of powdered *A. nilotica* leaf sample was extracted with 20% ethanol under gentle agitation. The extraction was repeated three times for consistency, and the combined extracts were evaporated to dryness. The total saponin content was calculated using Equation (2):

$Total saponin content \left( \% \right)= \left( \frac{WEP}{WPS} \right)*100$ (2)

where WEP = weight of the dried extract (end product) and WPS = initial weight of the powdered sample. This procedure provided a quantitative estimate of saponins, essential for assessing their contribution to the reducing and stabilizing potential of *A. nilotica* extracts in nanoparticle synthesis

**2.7S.1 UV–Vis Spectrophotometry**

UV–Vis spectra of ZnO and Fe_3_O_4_ nanoparticles were recorded using a UV–Vis spectrophotometer (Shimadzu UV-1800) in the wavelength range of 200–800 nm at a scan rate of 50 nm/min. Deionized water was used as the blank. For each analysis, 1.5 mL of the colloidal nanoparticle suspension was placed in a clean quartz cuvette. Samples were freshly prepared, cooled to room temperature, and analyzed immediately to prevent agglomeration.

Diffuse reflectance spectra were also recorded using an integrating sphere with BaSO_4_ as the reference. Reflectance (R) was converted to the Kubelka–Munk function using Equation (3):

$F(R)=\frac{(1-R)^{2}}{2R}$ (3)

The photon energy (*hν*) was calculated from the wavelength (λ) using *hν = 1240/λ (eV)*. Band gaps (*E₉*) were determined from Tauc plots based on *(αhν)¹/γ = A(hν − E₉)*, where γ = ½ for direct transitions and 2 for indirect transitions. The linear region of the plot near the absorption edge was extrapolated to *y = 0* to estimate *E₉*.

The UV–Vis spectrum of the *Acacia nilotica* extract was also recorded to identify and exclude background absorption bands (270–290 nm) arising from π→π* transitions of phenolic and flavonoid compounds. The band gap energy was calculated using Equation (4):

$E_{9}(eV)=\frac{1239.83}{\lambda}$ (4)

The calculated band gap energies for ZnO and Fe_3_O_4_ nanoparticles ranged between 2.50 and 4.27 eV.

**2.7S.2 Dynamic Light Scattering (DLS)**

Nanoparticle suspensions were prepared by dispersing ZnO and Fe_3_O_4_ powders in deionized water and sonicating for 10–15 min to minimize aggregation. The suspensions were filtered through a 0.22 µm membrane before measurement. DLS analyses were performed using a particle size analyzer equipped with a 633 nm laser at a 90° scattering angle. The hydrodynamic diameter, polydispersity index (PDI), and zeta potential were measured at 25°C. Each sample was analyzed in triplicate, and the results were averaged for precision.

**2.7S.3 High-Resolution Scanning Electron Microscopy (HRSEM) and Energy-Dispersive X-ray Spectroscopy (EDS)**

The surface morphology and microstructure of the nanoparticles were examined using a Zeiss Auriga high-resolution scanning electron microscope. Powder samples were mounted on carbon adhesive tape and sputter-coated with a thin layer of Au–Pd using a Quorum T15OT coater for 5 min to enhance conductivity. Images were obtained at an accelerating voltage of 15 kV. EDS was employed to determine elemental composition and confirm the purity and homogeneity of the synthesized nanoparticles.

**2.7S.4 X-ray Diffraction (XRD)**

Crystalline phases and average crystallite sizes of ZnO and Fe_3_O_4_ nanoparticles were determined using a Bruker AXS D8 Advance X-ray diffractometer with Cu Kα radiation (λ = 1.5406 Å). Diffraction data were collected over a 2θ range of 20°–90° with a step size of 0.02° and a counting time of 1 s per step. The obtained diffraction patterns were compared with Joint Committee on Powder Diffraction Standards (JCPDS) reference data for phase identification. The average crystallite size (***D***) was calculated using the Scherrer equation (Equation 5):

$D=\frac{0.9\lambda}{\beta\cos\theta}$ (5)

where *λ* is the X-ray wavelength (1.5406 Å), *β* is the full width at half maximum (FWHM) of the diffraction peak, and *θ* is the Bragg angle (in radians).

**2.7S.5 Brunauer–Emmett–Teller (BET) Surface Area Analysis**

Specific surface area, pore volume, and pore size distribution were determined using the BET nitrogen adsorption–desorption technique (Micromeritics ASAP 2020 analyzer). Before analysis, approximately 100 mg of ZnO and 50 mg of Fe₃O₄ powders were degassed under vacuum at 200°C (ZnO for 20 min; Fe₃O₄ for 6 h) to remove moisture and volatile impurities [27, 28]. Measurements were conducted at –196°C using liquid nitrogen. Surface area and pore characteristics were derived from BET plots correlating the adsorbed gas volume with relative pressure [29].

**2.7S.6 Fourier-Transform Infrared Spectroscopy (FTIR)**

FTIR spectra were obtained using a PerkinElmer 2000 spectrometer equipped with a DTGS detector over the range of 400–4000 cm⁻¹. Samples were prepared by mixing 0.01 g of dried nanoparticle powder with 200 mg of spectroscopic-grade KBr to form transparent pellets. The instrument chamber was purged with nitrogen gas to eliminate interference from moisture and CO_2_. The spectra were baseline-corrected and smoothed before analysis to identify the characteristic functional groups associated with metal–oxygen bond formation and organic capping agents from the *A. nilotica* extract.


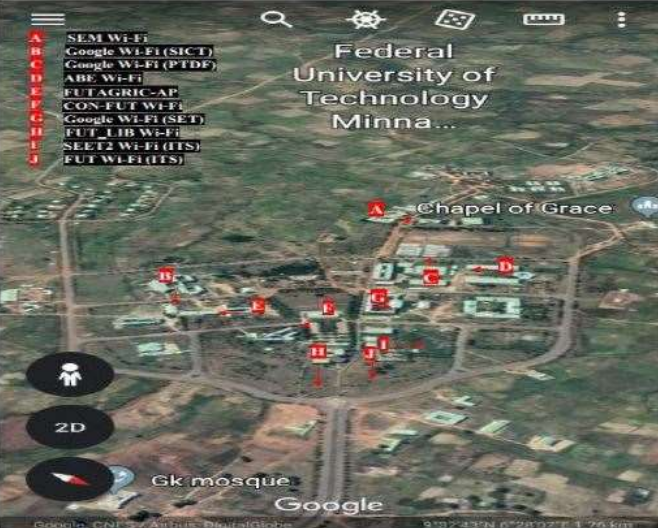


**Fig. S1: Google map of the study area**

1. Photoexcitation

$$ZnO/{Fe}_{3}O_{4}+hv\to e_{CB}^{-}+h_{VB}^{+} 6$$

2. Charge separation/transfer (synergistic effect)

$$e_{ZnO}^{-}\to h_{Fe_{3}O_{4}}^{-} 7$$

3. Reactive oxygen species (ROS) species generation

$$e_{CB}^{-}+O_{2}\to\cdot O_{2}^{-} 8$$

$$\cdot O_{2}^{-}+ H^{+}\to\cdot{HO}_{2} 9$$

$$2\cdot{HO}_{2}\to H_{2}O_{2}+O_{2} 10$$

4. $h_{VB}^{+}+H_{2}O\to\cdot OH+H^{+} 11$

$$h_{VB}^{+}+OH^{+}\to\cdot OH 12$$

5. Polyethylene chain activation

$$-CH_{2}-CH_{2}-+\cdot OH\to-CH_{2}-\cdot CH-+H_{2}O 13$$

6. Peroxy radical formation

$$-CH_{2}-CH_{2}-+O_{2}\to-CH_{2}-CHOO 14$$

7. Hydroperoxide formation

$$-CH_{2}-CHOO .+H^{+}\to-CH_{2}-CHOOH 15$$

8. Chain scission and oxidation

$$CH_{2}-COOH\to-CH_{2}-CHO-CH_{2}\cdot+\ldots16$$

9. Formation of low-molecular-weight products

$$Polymer fragments\to alcohols+aldehydes+carboxylic acids 17$$
